# Insights into the Evolution of a Snake Venom Multi-Gene Family from the Genomic Organization of *Echis ocellatus* SVMP Genes

**DOI:** 10.3390/toxins8070216

**Published:** 2016-07-12

**Authors:** Libia Sanz, Juan J. Calvete

**Affiliations:** Laboratorio de Venómica Estructural y Funcional, Instituto de Biomedicina de Valencia, Consejo Superior de Investigaciones Científicas, Jaume Roig 11, 46010 València, Spain

**Keywords:** Snake venom toxin multi-gene family, snake venom metalloproteinase, genomic organization of SVMP genes, PII-SVMP, PI-SVMP, gene duplication, intronic retroelements, intronization

## Abstract

The molecular events underlying the evolution of the Snake Venom Metalloproteinase (SVMP) family from an A Disintegrin And Metalloproteinase (ADAM) ancestor remain poorly understood. Comparative genomics may provide decisive information to reconstruct the evolutionary history of this multi-locus toxin family. Here, we report the genomic organization of *Echis ocellatus* genes encoding SVMPs from the PII and PI classes. Comparisons between them and between these genes and the genomic structures of *Anolis carolinensis* ADAM28 and *E. ocellatus* PIII-SVMP EOC00089 suggest that insertions and deletions of intronic regions played key roles along the evolutionary pathway that shaped the current diversity within the multi-locus SVMP gene family. In particular, our data suggest that emergence of EOC00028-like PI-SVMP from an ancestral PII(e/d)-type SVMP involved splicing site mutations that abolished both the 3′ splice AG acceptor site of intron 12* and the 5′ splice GT donor site of intron 13*, and resulted in the intronization of exon 13* and the consequent destruction of the structural integrity of the PII-SVMP characteristic disintegrin domain.

## 1. Introduction

The ADAM (A Disintegrin-like And Metalloproteinase) family of transmembrane type 1 proteins belongs to the MEROP database M12 family of Zn^2+^-dependent metalloendopeptidases [1] and PFAM family PF01421 [2]. Members of the ADAM family play important roles in cell signaling and in regulating cell-cell and cell-matrix interactions [3,4]. The ADAM family comprises ancient proteins whose origin extends back >750 My [5,6]. To date, close to 40 ADAM genes have been identified in vertebrate and invertebrate bilaterian animals, both in deuterostomes, from the basal chordate, *Ciona intestinalis*, to higher vertebrates, and in protostome, such as arthropods, nematodes, platyhelminths, rotifers, molluscs, and annelids. The evolutionary history of vertebrate ADAM genes is punctuated by gene duplication and retroposition events [7,8], followed by neo- or subfunctionalization [7]. Gene duplications are an essential source of genetic novelty that can lead to evolutionary innovation if the new function has no deleterious effects to its host organism or provides selective advantages. For example, in mammalian species, including marsupials and monotremes, except the platypus, ADAM28, ADAMDEC1 (decysin, a soluble ADAM-like protein), and ADAM7 form a cluster, likely as a result of tandem duplication of ADAM28 [9]. Instead, in most non-mammalian vertebrate genomes investigated, including those of aves, reptiles, and fishes, a single ADAM28 locus is present in this region [7,10]. The data suggest that ADAM7 and ADAMDEC1 were duplicated from ADAM28, probably only in mammals [7]. On the other hand, as described below in more detail, it is thought that ADAM28 played a starring role in the emergence of toxic metalloproteinases in the superfamily Colubroidea of Caenophidian snakes (viperids, elapids, and colubrids).

The concept that gene duplication plays a major role in evolution has been around for over a century [11]. In his classic and influential book “*Evolution by Gene Duplication*” [12] Susumo Ohno argued that gene duplication is the most important evolutionary force since the emergence of the universal common ancestor. Common sources of gene duplications include ectopic homologous recombination, retrotransposition event, aneuploidy, polyploidy, and replication slippage [13]. Duplication creates genetic redundancy, where the second copy of the gene is often free from selective pressure. Thus, over generations of the organism, duplicate genes accumulate mutations faster than a functional single-copy gene, making it possible for one of the two copies to develop a new and different function. Duplicated genes may switch their transcription to other tissues by localizing closely to, and utilizing the regulatory elements of, a neighboring gene [14,15,16]. Examples of this are (i) the formation of toxin gene families during the evolution of the venom system of advanced snakes by co-option, multiplication, and weaponization in the venom gland of paralogs of genes encoding for normal body proteins [17,18,19,20], and (ii) the finding of 309 distinct widow spider genes exhibiting venom gland biased expression [21], suggesting that the switching of genes to venom gland expression in numerous unrelated gene families has been a dominant mode of evolution [21,22,23].

Because of its functional importance for prey capture, predator defense, and competitor deterrence, venom represented a key innovation that has underpinned the explosive radiation of toxicoferan reptiles in the Late Jurassic period of the Mesozoic era, ~150 million years before present (MYBP) [24,25,26,27,28]. Toxicofera [18] (Greek for “those who bear toxins”) is the term coined for the clade of squamate reptiles that includes the Serpentes (snakes), Anguimorpha (monitor lizards, gila monster, and alligator lizards) ,and Iguania (iguanas, agamas, and chameleons) lizards. One of the founding families of advanced snake venom comprises the Zn^2+^-dependent metalloendopeptidases (SVMPs) [17,18,19,29,30,31,32]. SVMPs are key enzymes contributing to toxicity of vipers and pitvipers venoms. Hemorrhage is one of the most significant effects in envenomings induced by viperid and crotalid snakebites. Damage to the microvasculature, induced by SVMPs, is the main event responsible for this effect. In addition to hemorrhagic activity, members of the SVMP family also have fibrin(ogen)olytic activity, act as prothrombin activators, activate blood coagulation factor X, possess apoptotic activity, inhibit platelet aggregation, are proinflammatory, and inactivate blood serine proteinase inhibitors [33,34,35,36].

**Figure 1:**
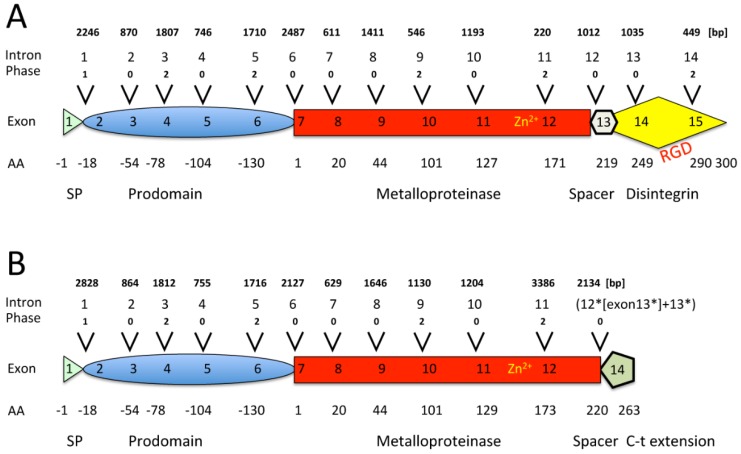
Scheme of the genomic organization of pre-pro EOC00006-like PII-SVMP (**A**) and pre-pro EOC00028-like PI-SVMP (**B**) genes. The distribution, phase, and size of the 14 (PII) and 12 (PI) introns and the boundaries of the protein-coding regions are highlighted. SP, signal peptide. Homologous exons and introns have identical numbering. Intron 12 of the PI-SVMP gene corresponds to the fusion of the genomic segment spanning intron12*-exon13*-intron13*. Mature PII- and PI-SVMP amino acid sequences span 299 and 263 amino acid residues, respectively. Zn^2+^, relative location of the catalytic Zn^2+^-binding environment; RGD, integrin-binding arginine-glycine-aspartic acid tripeptide motif.

The closest non-venom ancestors of SVMPs was likely an ADAM28 precursor gene [37]. The origin of SVMPs has been inferred to have occurred following the split of the Pareatidae from the remaining Caenophidians, approximately 60 MYBP around the Cretaceous–Paleocene boundary of the Cenozoic Era [18,19,29,31,38]. SVMPs are found in the venoms of all advanced snakes and are classified into different classes depending upon their domain structure [39,40,41]. The ancestral multidomain PIII form, which is found in all snake venoms, derives from the extracellular region (metalloproteinase domain with disintegrin-like and cysteine-rich domains at the *C*-terminus) of a duplicated ADAM28 precursor gene that lost the *C*-terminal epidermal-growth-factor (EGF-)-like, transmembrane, and cytoplasmic domains [31,32,41,42,43]. On the other hand, the derived PII-SVMPs, comprising the metalloproteinase and *C*-terminal disintegrin domain, have been only found in venoms of vipers and rattlesnakes (Viperidae). This strongly suggests that they emerged, subsequently to the separation of Viperidae and Elapidae, ~37 million years ago, in the Eocene epoch of the Cenozoic era, but before the separation of the Viperidae subfamilies Viperinae and Crotalinae 12–20 MYBP, from a duplicated PIII-SVMP gene that lost its cysteine-rich domain (see Figures 1 and 8 in [43] and Figure 18.1 in [44]). The disintegrin domain has been lost from the PII-SVMP structure on multiple occasions, resulting in the formation of the PI class of SVMPs [45] made only by the catalytic Zn^2+^-metalloproteinase domain [39,40,41].

Details on the mechanisms of co-option and the molecular events underlying the transformation of an ADAM28 precursor gene copy into the SVMP multi-gene family of extant snake venoms remain elusive. In previous works, we described a family of RPTLN genes that exhibit a broad and reptile-specific distribution, for which we hypothesize may have played a key role in the recruitment and restricted expression of SVMP genes in the venom gland of Caenophidian snakes [46]. We have also reported the genomic organization of *Echis ocellatus* PIII-SVMP gene EOC00089, and compared it to those of its closest orthologs from *Homo sapiens* and the lizard, *Anolis carolinensis* [47]. Now, we fit two new pieces in the puzzle: the genomic structures of *E. ocellatus* PII—(EOC00006-like) and PI—(EOC00028-like) SVMP genes. Insights into post-duplication events gained from the structural comparison of the three classes of SVMP genes are discussed.

## 2. Results and Discussion

### 2.1. The Genomic Structure of Pre-Pro EOC00006-Like PII-SVMP and Pre-Pro EOC00028-Like Genes

**Figure A1:**
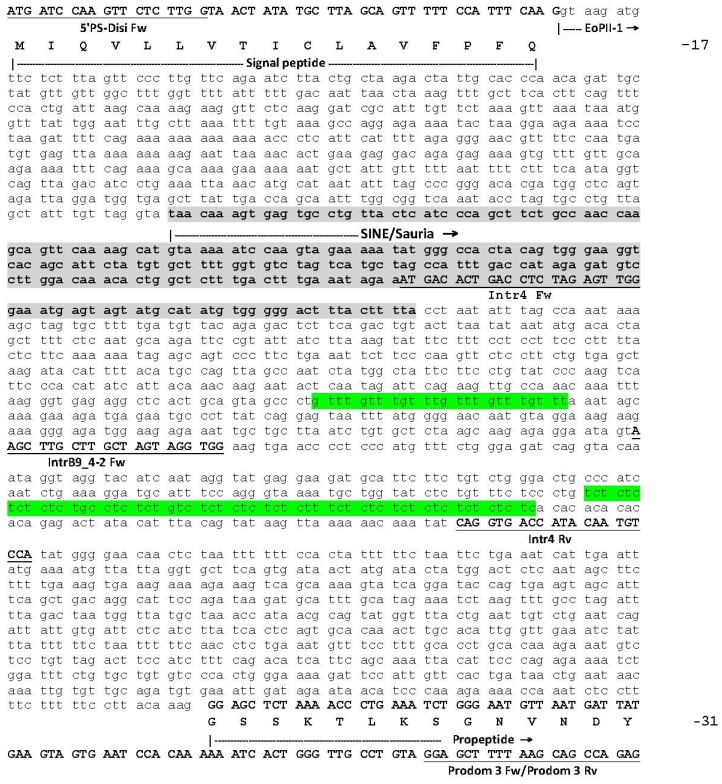

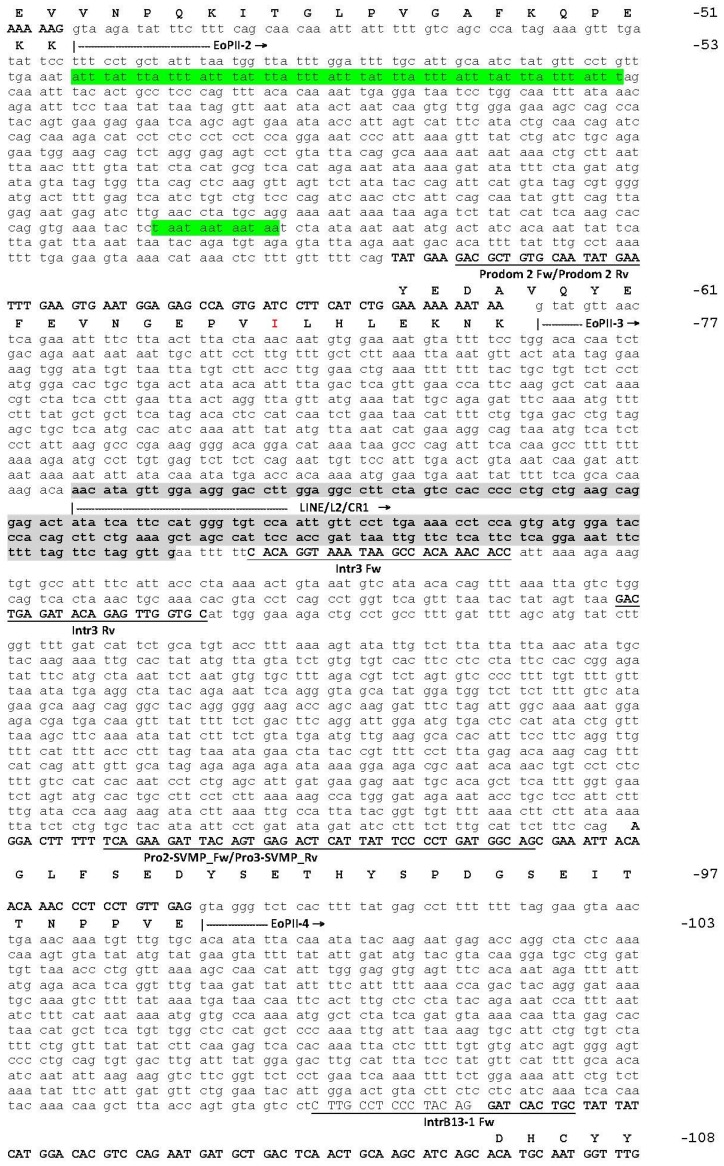

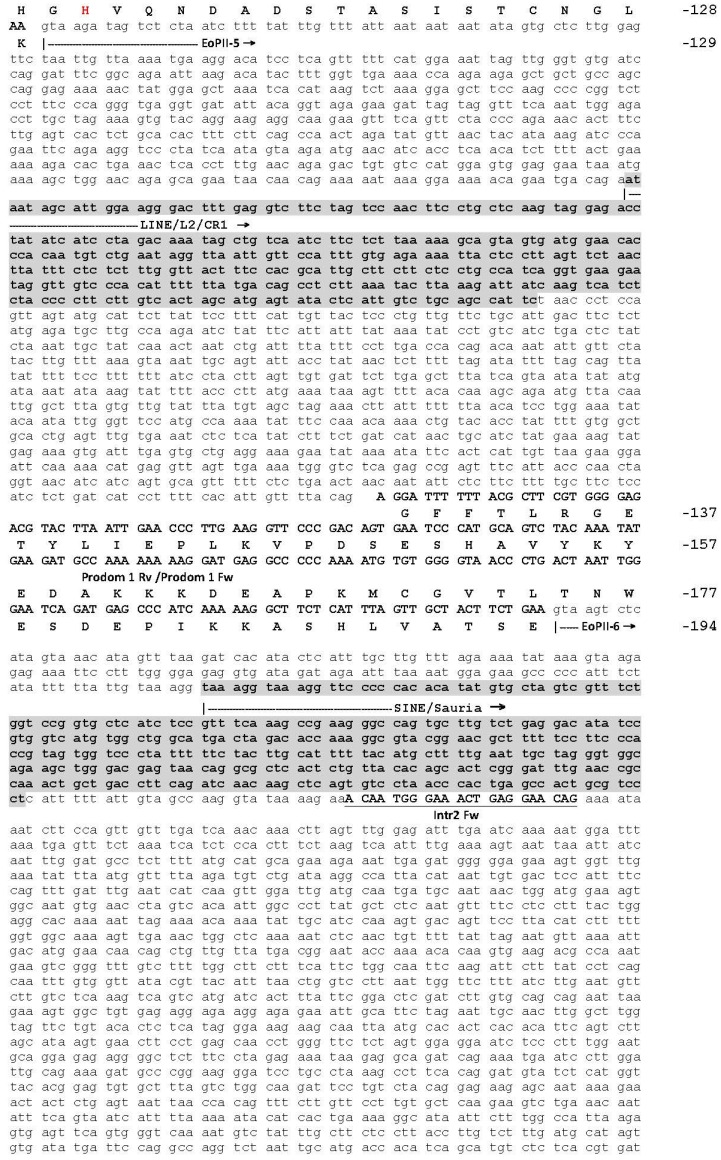

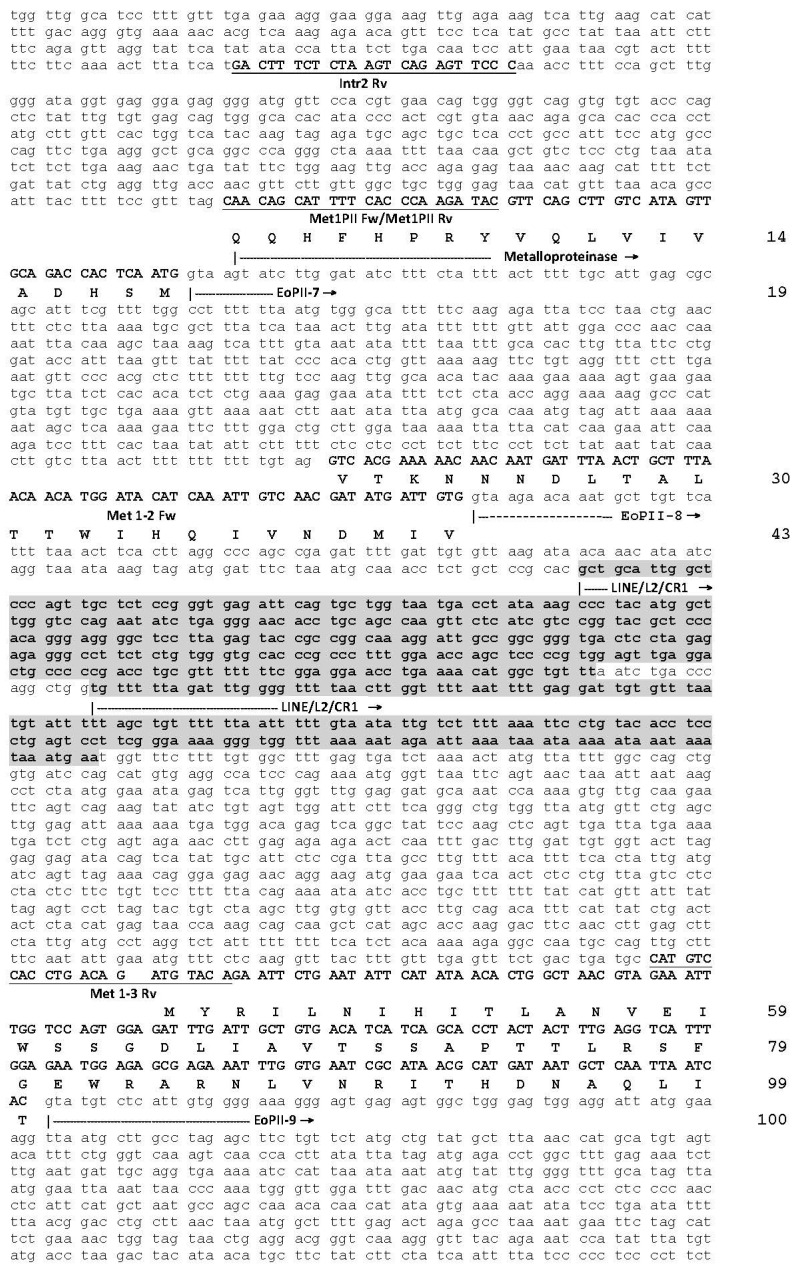

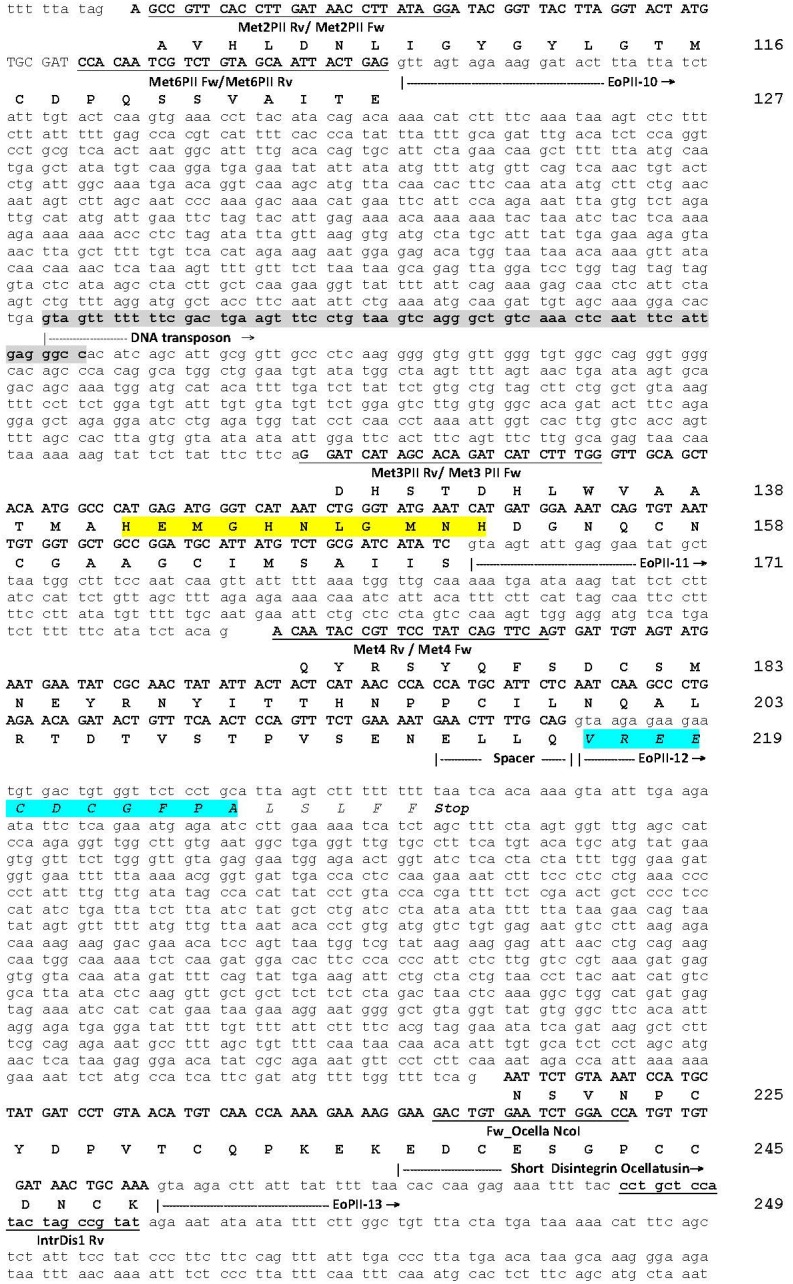

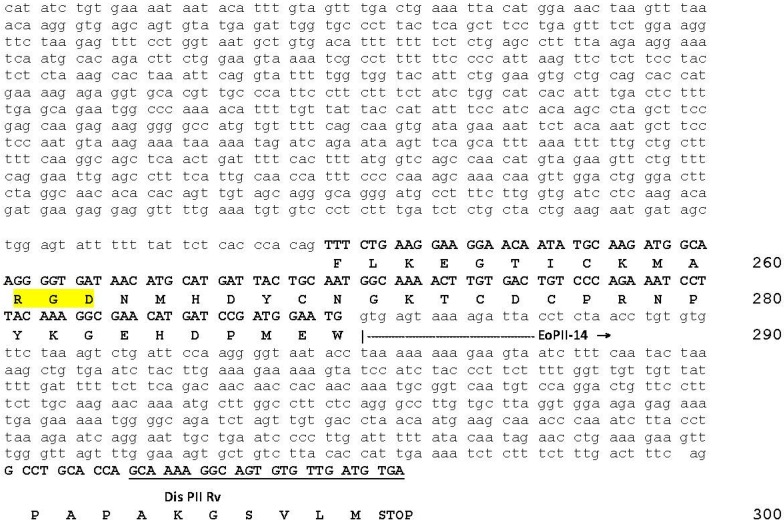
Genomic organization of *E. ocellatus* EOC00006-like PII-SVMP gene.

**Figure A2:**
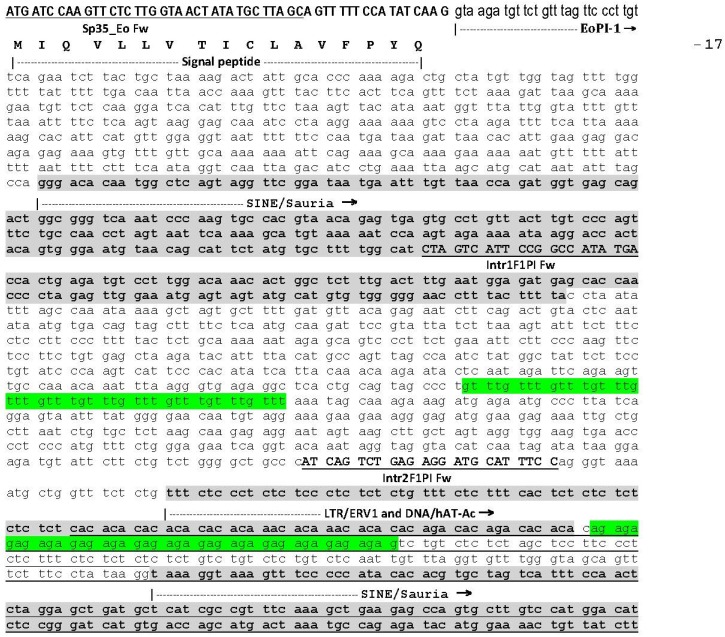

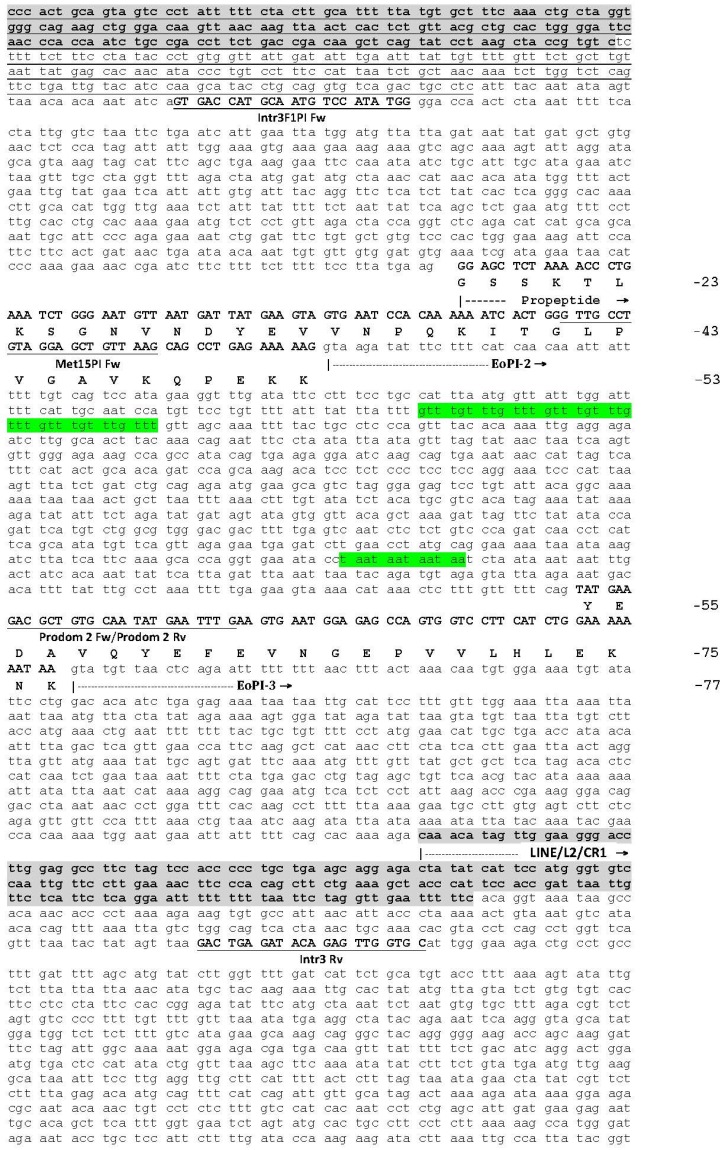

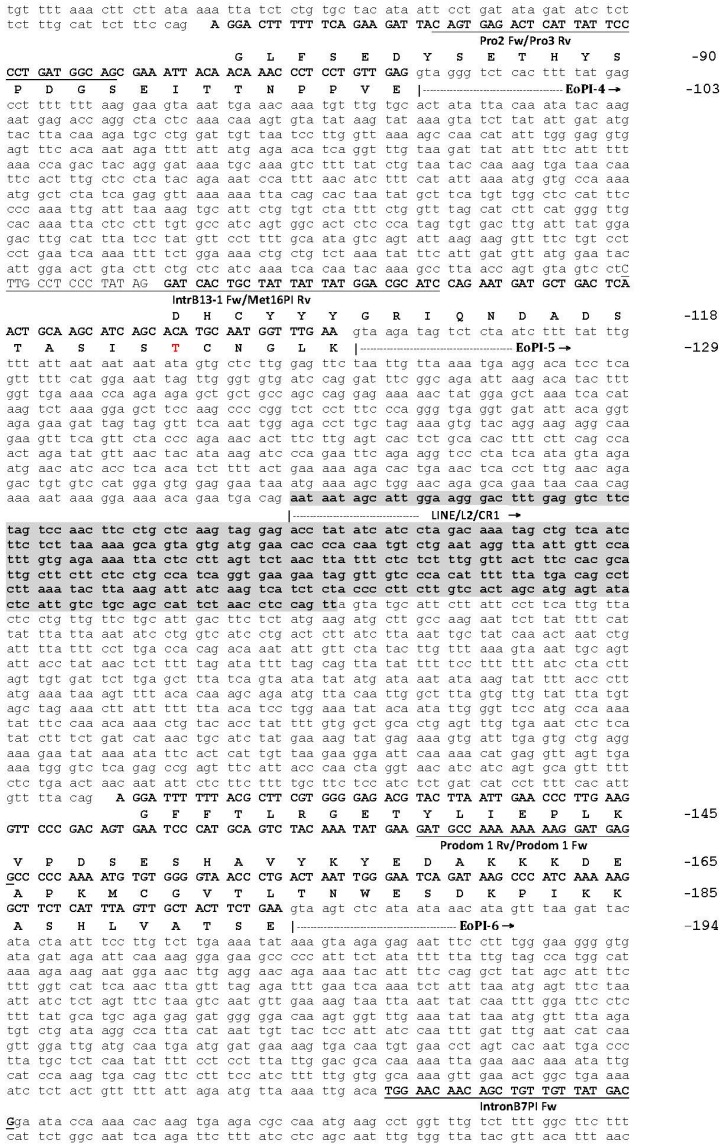

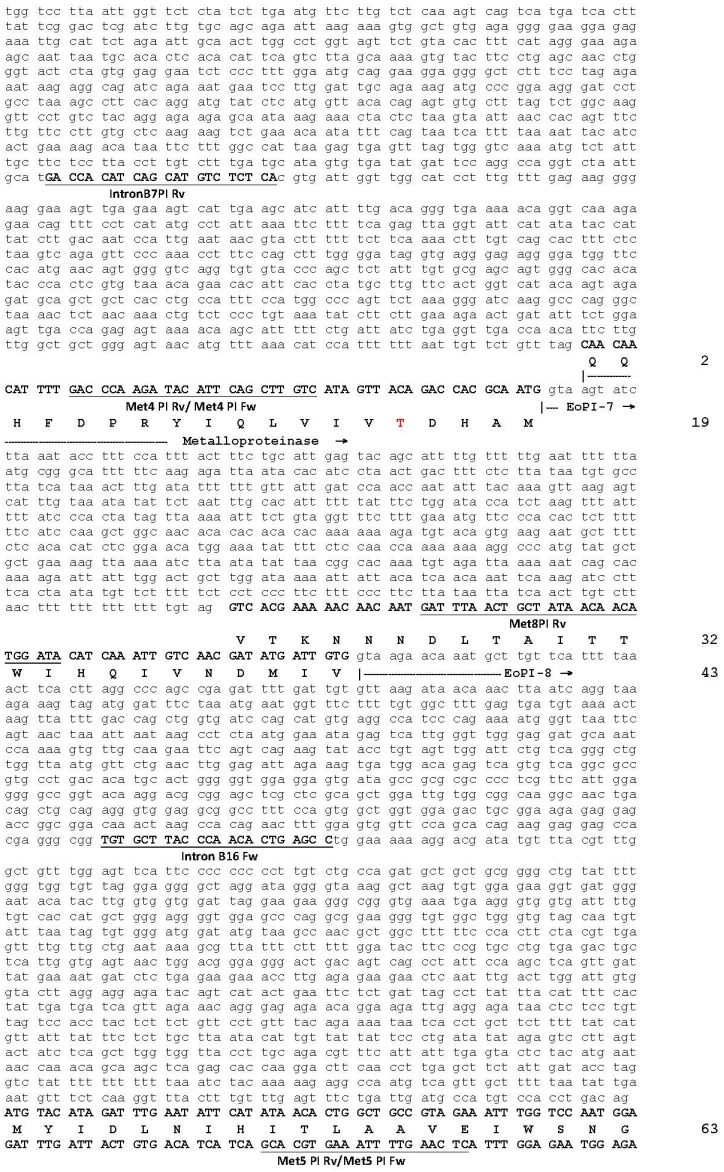

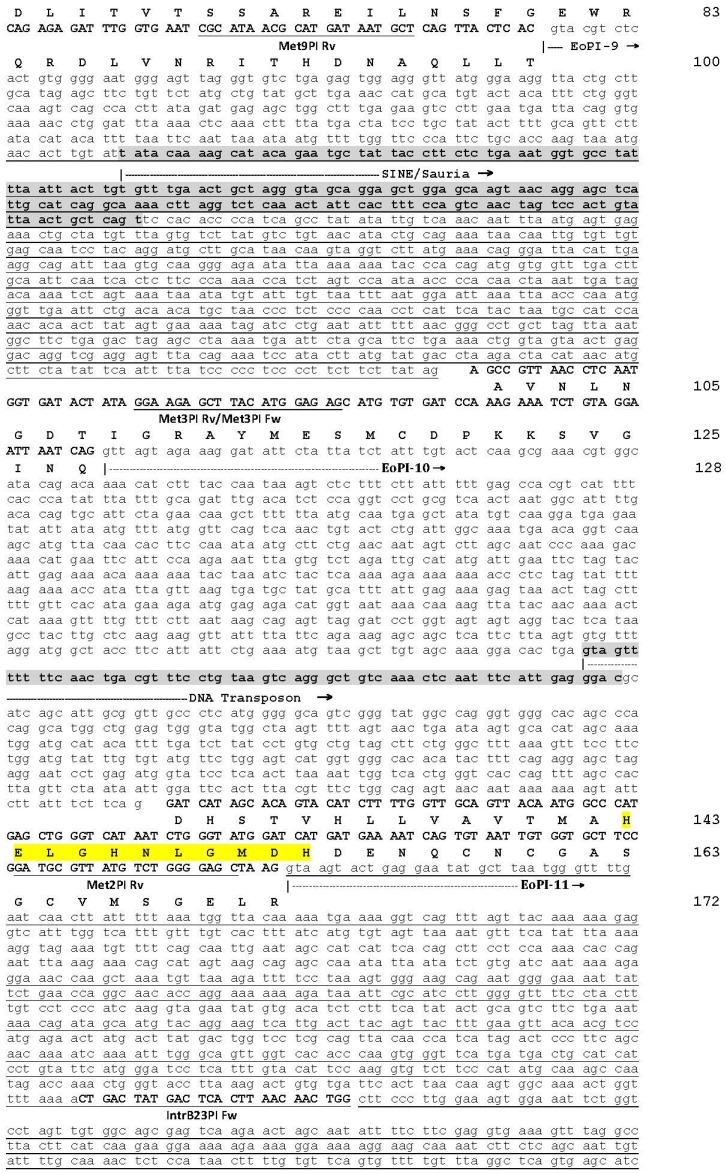

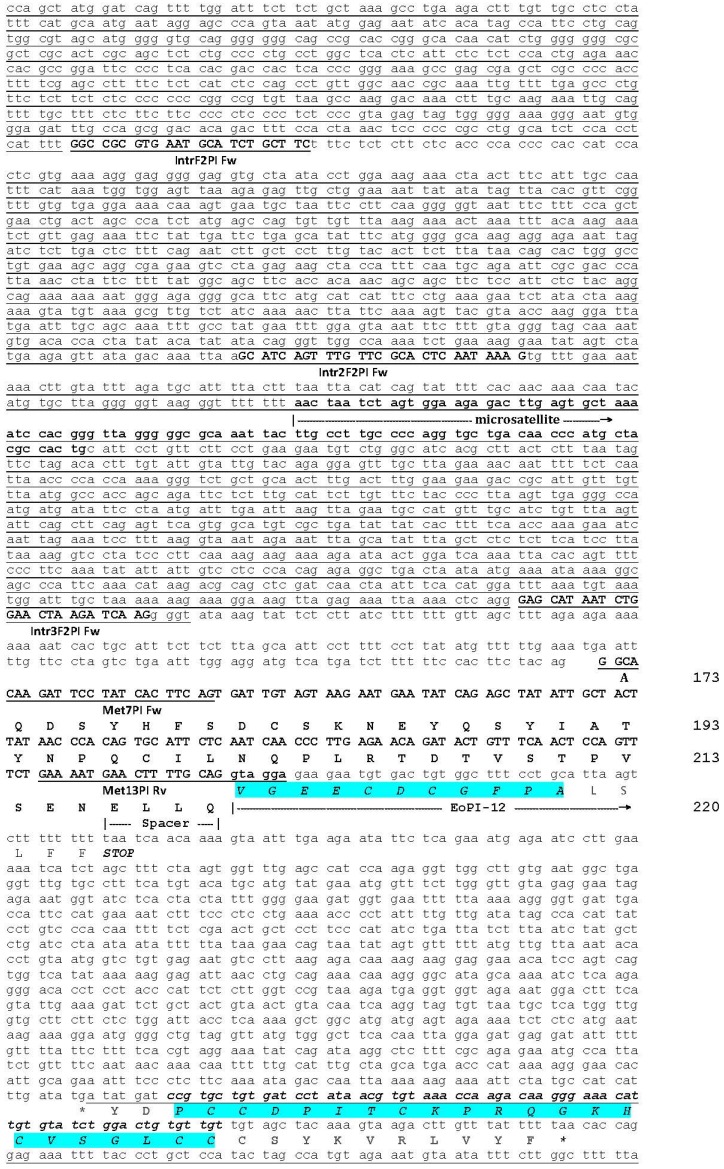

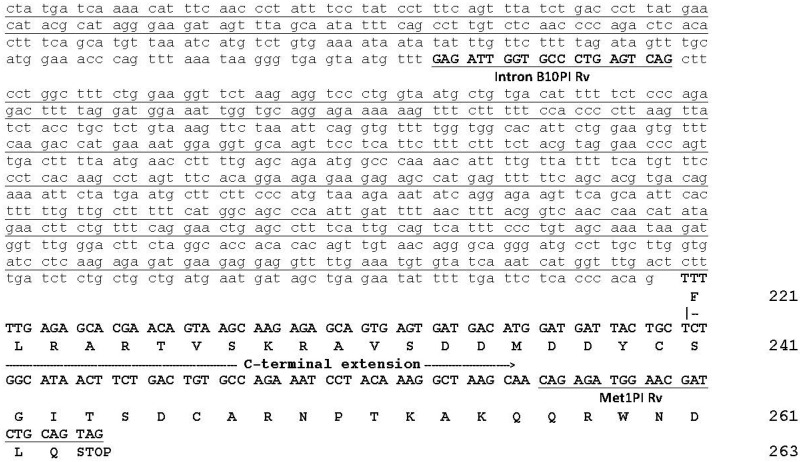
Genomic organization of *E. ocellatus* EOC00028-like PI-SVMP gene.

Genomic sequences encoding full-length pre-pro EOC00006-like PII-SVMP (17828 nt) [KX219964] (Figure A1) and EOC00028-like PI-SVMP (21605 nt) [KX219965] (Figure A2) genes were assembled from overlapping PCR-amplified fragments (Appendix A, Figure A1 and Figure A2). The pre-pro PII-SVMP gene consists of 15 exons interrupted by 14 introns (Figure 1A), whereas the pre-pro PI-SVMP gene contains 13 exons and 12 introns (Figure 1B).

**Figure 2:**
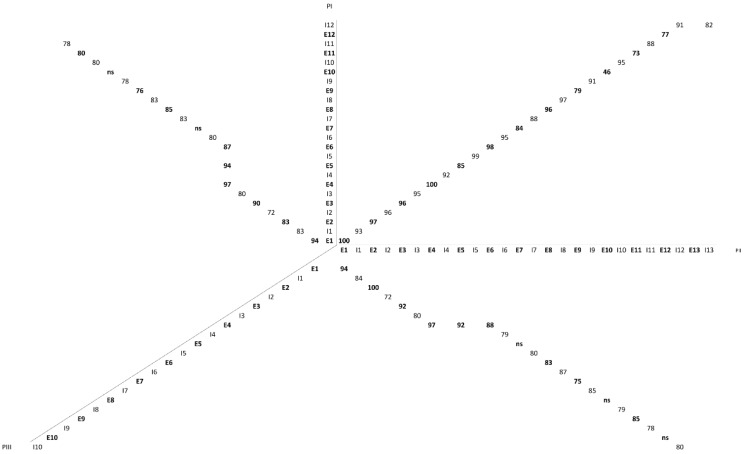
Pairwise comparisons of the sequence identities between the exonic and intronic nucleic acid sequences of pre-pro EOC00089-like PIII-SVMP, EOC00006-like PII-SVMP, and EOC00028-like PI-SVMP genes.

The translated 494 (PII) and 457 (PI) pre-pro-SVMP amino acid sequences exhibit identical distribution and features (in terms of codon location and phase) for their first 11 introns and 12 exons, which code for the signal peptide (SP), prodomain (PD), metalloproteinase (MP) domain, and the short tetrapeptide (ELLQ) “spacer” sequence (Appendix A, Figure A1 and Figure A2). These 413 (PII)/414 (PI) amino acid sequences show 85% identity, strongly suggesting that both SVMPs have a shared ancestry. It is also worth noting that the protein-coding positions interrupted by each of the introns of the PII- and PI-SVMP genes are entirely conserved in *Anolis carolinensis* [XP_008118058] (and also in human [NG_029394]) ADAM28 gene. Introns are inserted after or between secondary structure elements, supporting the “introns-added-late” model, which proposes that during the evolution of the eukaryotic branch, introns were added at the boundaries of structural modules coded for by ancestral continuous genes [48]. In addition, as will be analyzed in detail below, pairwise alignment of topologically equivalent PII- and PI-SVMP introns show that homologous intronic nucleic acid sequences share 88%–99% identity (Figure 2). This clearly indicates that EOC00006-like PII-SVMP and EOC00028-like PI-SVMP represent paralog genes.

New genes can arise through four mechanisms: gene duplication, retroposition, horizontal gene transfer, and de novo origination from non-coding sequences [49]. Available evidence strongly suggests that gene duplication has played a pivotal role in the origin of venom multi-gene families [20,21,22,23,50,51]. Although the fate of many new genes may be to lose their function and become pseudogenes, some can be fixed through evolution of redundancy, subfunctionalization, or neofunctionlization. Several models have been proposed to explain functional divergence following venom toxin gene duplications [52,53,54,55]. However, this issue remains controversial and is the subject of vivid debates. The family portrait of SVMPs shows a complicated picture. SVMPs belong to different “generations”, that in the canonical model for the evolutionary expansion of this multi-gene family are hierarchically related, being PIII-SVMPs the most ancient and the PII- and PI- SVMPs the succesively most recently derived family members [31,32,42]. However, due to the limited genomic information available, this model can be confounded by high rates of protein amino acid sequence divergence [56], and the occurrence of alternative routes (e.g., PIII > PI) can not be presently ruled out. The only other full-length viperid SVMP gene sequenced to date is *E. ocellatus* EOC00089-like PIII-SVMP [47] [KX219963]. The ORF encoding the pre-pro-metalloproteinase domains of this gene exhibits 63% amino acid sequence identity with the homologous coding regions of the PII- and PI-SVMPs here reported, and 72%–83% nucleotide sequence identity between topologically equivalent PIII-, PII-, and PI-SVMP introns (Figure 2). Although these figures clearly point to a common origin, it is not possible to infer whether they belong to the same or to a different PIII > PII > PI hierarchical lineage. Nonetheless, the fact that the PIII-SVMP gene has lost introns 5 and 6 (ADAM28 numbering), with the consequence that exons 4, 5, and 6 have merged into a single exon, suggests that either these events occurred after the duplication that gave rise to the PII-SVMP ancestor, or that the PIII-SVMP EOC00089-like gene does not lay in the direct line of descent of the EOC00006-like PII-SVMP and EOC00028-like PI-SVMP genes. Refinement of the family tree of the multi-gene family of *E. ocellatus* SVMPs will surely emerge from future comparative genomic analysis of the carpet viper and other viperid species.

### 2.2. Role of Introns in the Evolution of the SVMP Multi-Gene Family

Since their discovery in 1977 [57,58], introns have been the subject of considerable debate. It is now generally accepted that introns represent more than merely junk DNA that must be pruned from pre-mRNAs to yield mature, functional mRNAs prior to their translation. Mounting evidence indicates that while introns do not encode protein products, they play essential roles in a wide range of gene expression regulatory functions such as non-sense mediated decay [59], mRNA export [60], and regulation of the amount of recombination between the flanking exons [61], or they serve as locations for nonhomologous recombination that would allow for exon shuffling [62,63]. As discussed below, most of the structural divergence between the EOC00006-like PII-SVMP and EOC00028-like PI-SVMP genes is due to the different size of their topologically equivalent eleven (1–11) introns (Supplementary Figure S1). The role of introns in the evolution of snake venom gene families remains elusive. However, in other biological systems, i.e., *Arabidopsis* and *Drosophila*, intron features, such as sequence and length, have been shown to function in maintaining pre-mRNA secondary structure, thus influencing temporal and spatial patterns of gene expression by modulating transcription efficiency and splicing accuracy [64,65,66,67].

Most PII- and PI-SVMP introns belong to phase 0, followed by phase 2; and, in both genes, only intron 1, separating the monoexonic signal peptide from the start of the prodomain, is a phase 1 intron (Figure 1). Analysis of the exon–intron structures of a large number of human genes has revealed a statistically highly significant enrichment of phase 1 introns flanking signal peptide cleavage sites [68]. Phase 1 introns most frequently split the four GGN codons encoding glycine. A plausible explanation for the correlation between signal peptide domains and the intron phase is that the base preferences of proto-splice sites [69,70] mirrors the amino acid preference for glycine in the signal peptidase consensus cleavage site [71].

The signal peptide is the most conserved structural element between pre-pro EOC00006-like PII-SVMP and EOC00028-like PI-SVMP is (Figure 2). In both genes, it is encoded by identical exon 1 amino acid sequences (Figure A1 and Figure A2), which is also highly conserved in present-day SVMPs [46]. These findings support the view that co-option of this signal peptide may have played a role in the restricted expression of SVMP genes in the venom gland of Caenophidian snakes, some 60–50 Mya [46].

Nucleotide sequence comparison of the topologically equivalent introns of the *E. ocellatus* PII- and PI-SVMPs (Supplementary Figure S1) provide insights into the events underlying the conversion of a PII-SVMP into a PI-SVMP gene. In this regard, some introns differ in the number and location of intronic retroelements (Table 1). Thus, insertions in introns PI-SVMP 1 and 9 introduced complete and truncated SINE/Sauria elements in positions 1764–2101 (Figure S1, panel A) and 321–502 (Figure S1, panel I), respectively. The inserted nucleic acid sequence in intron 9 retains the GT-AG splicing sites, indicating that this insertion event created a twintron, an intron within an intron. PII-SVMP intron 6 (Figure S1, panel F) and PI-SVMP introns 11 (Figure S1, panel K) and 12 (Figure S1, panel L) are also twintrons. Compared to its topologically equivalent PII-SVMP intron, a large insertion in intron 11 of the EOC00028-like PI-SVMP gene replaced the first 66 nucleotides for a longer stretch of 3281 nucleotides; region 2461–2561 of the inserted nucleic acid sequence is 97% identical to *Hyla tsinlingensis* Hts-35 [KP204922], a microsatellite sequence that is also partly present in intron 61 of Podarcis reelin (RELN) genes [GU181006-13] (positions 554–623) [72]. Microsatellites are simple nucleotide sequence repeats (SSR) ranging in length from two to five base pairs that are tandemly repeated, typically 5–50 times (reviewed in [73]). These non-coding elements are abundant in major lineages of vertebrates. Mammalian, fish, and squamate reptile genomes appear to be relatively microsatellite rich [74]. However, besides Hts35, RepeatMasker only identified few SSR tracks in introns 1 (5× GTTT; 28× TC) and 2 (13× ATTT; 4× TAA) of the PII-SVMP gene (Figure A1), and introns 1 (11× GTTT; 21× AG) and 2 (9× GTTT; 4× TAA) of the PI-SVMP gene (Figure A2).

Growing evidence supports that repetitive intronic elements, such as the long interspersed elements (LINEs) and the short interspersed elements (SINEs) contained in several introns of both PII- and PI-SVMP genes (Table 1) can influence genome stability and gene expression (reviewed in [75]). Thus, these interspersed repeats may alter genome recombination structure and rates, through a number of mechanisms, including replication slippage and unequal crossover [76,77], potentially impacting regulation of gene expression [78], recombination events leading to tandem duplication of segments of the genome [79,80], gene conversion [81], and chromosomal organization [79]. Moreover, the insertion of interspersed repeats into a new genomic position may introduce promoter or enhancer sequence motifs for transcription of nearby genes [82,83], and alternative splicing sites or polyadenylation sites [84], thereby resulting in a change of overall level of gene expression. Interspersed repetitive elements have also played an important role in expanding the repertoire of transcription factor binding sites in eukaryotic genomes [85]. However, whether these elements have contributed to the genomic context that facilitated the evolution and radiation of venom *loci* in snakes deserves future detailed comparative genomic studies.

### 2.3. A Fusion Event Led to the Conversion of a PII(e/d)-Type SVMP into EOC00028-like PI-SVMP

**Figure 3:**
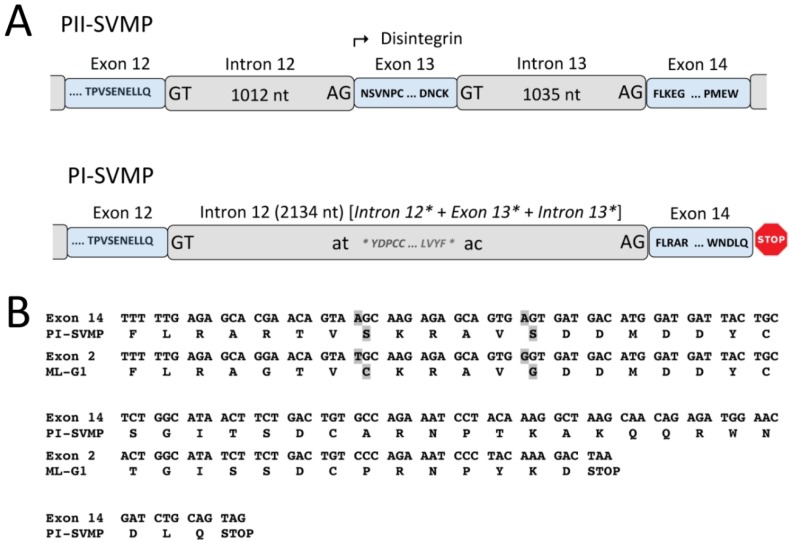
Panel **A**, cartoon comparing the 3′ regions of the PII-SVMP and PI-SVMP genes and highlighting the processes (intronization of ancestral exon 13* inside twintron 12 resulting from the fusion of introns 12* and 13*, and creation of a stop codon after exon 14) that destroyed the integrity of the disintegrin domain, converting an ancestral PII(e/d)-type SVMP into extant EOC00028-like PI-SVMP. Panel **B**, alignment of the amino acid sequences encoded by exon 14 of EOC00028-like PI-SVMP and exon 2 of the dimeric disintegrin subunit ML-G1 [AM261811] [87]. Degeneration of PI-SVMP’s conserved functional and structural amino acid residues in dimeric disintegrins are highlighted in boldface and grey background.

PI-SVMP intron 12 is a twintron resulting from the fusion of the genomic region spanning ancestral introns 12* and 13* and exon 13* (homologous to identical numbered elements in the genomic structure PII) (Figure 1 and Figure 3A). Splicing site mutations affecting both the 3′ splice AG acceptor site of intron 12* and the 5′ splice GT donor site of intron 13* led to the retention, and subsequent intronization, of exon 13* within a fused (12* + 13*) twintron (Figure 3A). Intronization of exon-coding nucleic acid sequences has been proposed as a major contributor to intron creation [86]. Intron 13* encoded part of the *N*-terminal region of a disintegrin domain, most likely, as discussed below, an eventual subunit of dimeric disintegrin. In addition to the disruption of the structural integrity of the disintegrin domain, a stop codon after exon 14 removed intron 14 and exon 15 from the PII(e/d)-type SVMP (Fox & Serrano’s nomenclature [40]) precursor gene structure, thereby completing the conversion of the PII-SVMP into present EOC00028-like PI-SVMP gene (Figure 3A).

Region 1013–2134 of PI-SVMP intron 12 exhibits 91% nucleotide sequence identity with range 14 to 1135 of *Macrovipera lebetina* gene encoding part of exon 1 and full-length intron 1 of the VGD-containing dimeric disintegrin subunit precursor, ML-G1 [AM261811] [87]. PI-SVMP exon 14 (mature protein amino acid residues 221–263, Figure A2) exhibits strong homology (79% identity) to exon 2 of the same VGD-bearing dimeric disintegrin subunit. The PI-SVMP exon 14 shows the consequences of genetic drift (Figure 3B): the conseved α_5_β_1_ integrin-inhibitory VGD tripeptide motif [44] of the PII-SVMP precursor gene has been replaced by a VSD motif (generated by a G > A mutation: GTG AGT GAT > GTG GGT GAT), and the absolutely conserved tenth cysteine residue of dimeric disintegrin subunits has degenerated (TGC) to a serine residue (AGC) (Figure 3B).

## 3. Concluding Remarks and Perspectives

**Figure 4:**
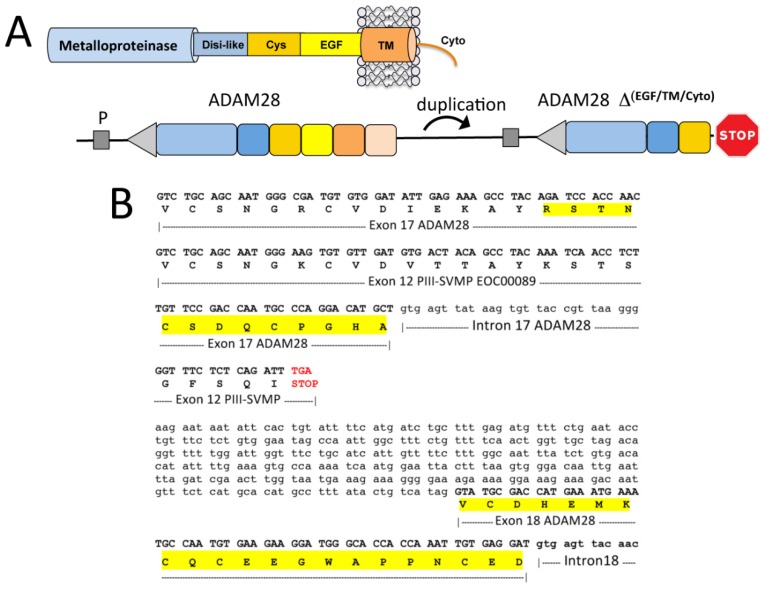
Comparison of the genomic region encompassing exons 17 through 18 of *Anolis carolinensis* ADAM28 [XP_003226913] and the homologous amino acid sequence of *E. ocellatus* SVMP EOC00089 [ADW54351], highlighting the STOP codon after exon 12 of the latter generating a *C*-terminally truncated molecule, which eventually gave rise to the ancestor of the PIII-SVMPs.

**Figure 5:**
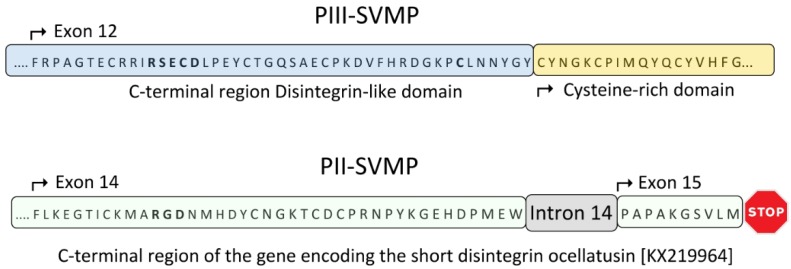
Comparison of the genomic region encoding the *C*-terminal domains of *E. ocellatus* EOC00089-like PIII-SVMP and EOC00006-like PII-SVMP, suggesting that 3′ genomic remodeling represents a seminal step in the generation of PII-SVMPs.

The event that gave birth to the family of SVMPs was the generation of a STOP codon at the 3′ end of exon 16 of a duplicated ADAM28 gene (Figure 4). This mutation produced an ORF truncated at the *N*-terminal part of the EGF-like domain, which encoded a precursor of an ancestral PIII-SVMP lacking this domain and the *C*-terminal membrane anchoring and cytoplasmic polypeptides (Figure 4). On the other hand, our results comparing the available genomic structures of SVMP genes, e.g., EOC00089-like PIII-SVMP [47] [KX219963], EOC00006-like PII-SVMP [KX219964], and EOC00028-like PI-SVMP [KX219965] (this work), suggest that the evolutionary history of SVMPs is marked with events of insertions and deletions of intronic regions. This scenario points to introns as key players in the formation of the multi-locus SVMP gene multifamily. Thus, comparison of the genomic structures of EOC00089-like PIII-SVMP and EOC00006-like PII-SVMP (Figure 5) indicates that replacement of the PIII-specific cysteine-rich domain by a non-homologous region encoding intron 14-exon 15 followed by a STOP codon may represent a step in the conversion of a PIII-SVMP into a PII-SVMP.

This view is consistent with structural evidence suggesting that the loss of the cysteine-rich domain represents an early seminal event that facilitated the formation of PII class SVMPs [43]. The PII subfamily of SVMPs is characterized by the diversity of disintegrin domains exhibited by different family members [39,40], ranging from the more ancestral long disintegrin domains (~84 amino-acid-residue polypeptide cross-linked by 7 disulfide linkages) to the more recently evolved short disintegrin (41–51 amino-acid-residues crosslinked by 4 disulfide bonds) [42]; for a scheme of the evolutionary path of the disintegrin domains, see Figure 1 in [43]. EOC00006-like is an example of a PII-SVMP with short disintegrin domain. Given the structural diversity of PII-SVMPs, genomic sequences from the different members of the subfamily are required for a more accurate glimpse of the genomic mechanisms operating in the generation and subsequent diversification of PII-SVMPs.

Comparison of the EOC00006-like PII-SVMP and EOC00028-like PI-SVMP gene structures also points to genomic remodeling of the 3′ region of a PII(e/d)-type SVMP precursor gene [39,40] as the EOC00028-like PI-SVMP gene generator mechanism. The PII > PI conversion involved the generation of twintron 12 (by fusion of introns 12* and 13*) and the loss, by intronization, of exon 13*, thereby destroying the consistency of the region coding for the disintegrin domain. This elaborated mechanism indicates that the structural diversification of SVMPs is not due to a random mutation generating a STOP codon before the disintegrin domain, but follows a well orchestrated sequence of events imprinted in the genome of snake species sometime after the split of Viperidae and Elapidae, 37 million years ago, but before the separation of the Viperidae subfamilies Viperinae and Crotalinae 12–20 MYBP. The mechanisms underlying loss or gain of spliceosomal introns are still poorly understood. The most widely accepted hypothesis is that intron insertion may occur via a process similar to group II intron retrotransposition [88,89]. According to this view, the spliceosomal components remain transiently associated with a recently excised intron and then attach at a potential splice site of a non-homologous pre-mRNA, where they catalyze the reverse reaction [90,91]. The modified pre-mRNA is reverse-transcribed and the resulting cDNA participates in a recombination with its parent gene, thereby inserting a novel intron into the target gene [90,91,92,93]. An attractive feature of this mechanism is that it ensures that the inserted nucleic acid sequence has the full complement of intron signature sequences required for efficient splicing [94].

Studies of multi-gene protein families are crucial for understanding the role of gene duplication and genomic exon-intron organization in generating protein diversity. For example, full-length genomic sequences of Crotalinae group II PLA_2_ isogenes from *P. flavoviridis* (Tokunoshima and Amami-Oshima islands, Japan) [95], and *T. gramineus* (Taiwan) [96] have been reported. All these genes exhibit four coding regions and conserved exon-intron structures spanning about 1.9 kb. A cluster of five tandemly arranged PLA_2_ genes have been located in a 25 kb 3′ segment of a 31 kb fragment of the Amami-Oshima *P. flavoviridis* genome [97], which in addition harbors a PLA_2_ pseudogene in its 6 kb 5′ region [98]. Genomic sequence comparisons between the pancreatic PLA_2_ gene of *P. elegans*, group IB pancreatic PLA_2_ gene of *L. semifasciata*, and the *L. semifasciata* group IA venom PLA_2_ gene, suggest that Crotalinae group II venom PLA_2_ genes emerged before the divergence of Elapinae and Crotalinae, whereas groups of IB and IA PLA_2_ genes appeared after Elapinae was established as a taxonomic lineage [99].

Duplicated structures found in eukaryotic genomes may result from complex interplays between different mechanisms [100]. Mitotic and meiotic non-allelic homologous recombination (NAHR) events, resolved as unequal crossing-over, have been traditionally invoked to account for segmental duplications within genomes [101,102]. Duplicated regions can be organized as direct tandems (e.g., the cluster of tandem snake venom PLA_2_ genes), but also be separated by hundreds of kb [100]. Our present and previous work [47] inaugurate a line of research that will allow the depiction of a more precise characterization of the genomic context in which the SVMP multi-gene family has emerged. This goal demands populating the current databases with genomic sequences of genes representing the different members of the SVMPs. Although the variety of structural forms comprising the PII family may be considered a challenge for this purpose, this circumstance can be also regarded as a valuable opportunity for the step-by-step description of the molecular pathways that led to the formation of this multi-gene family. Without a doubt, ongoing Viperidae snake genome sequencing projects will mark the beginning of comparative snake genomics, and will be key to revealing not only the topology and copy number of the genes encoding SVMPs, but also to provide decisive information to reconstruct the evolutionary history of this multilocus gene family.

## 4. Materials and Methods

### 4.1. Genomic DNA

Genomic DNA was extracted from the fresh liver of *E. ocellatus* (Kaltungo, Nigeria) maintained at the herpetarium of the Liverpool School of Tropical Medicine. *Echis ocellatus* liver was ground to a fine powder under liquid nitrogen and the genomic DNA extracted using a Roche DNA isolation kit for cells and tissue containing SDS (2% final concentration) and proteinase K (400 μg/mL final concentration). The homogenates were incubated at 55 °C overnight. Thereafter, 300 μL of 6 M NaCl (NaCl-saturated H_2_O) was added to each sample, and the mixture was vortexed for 30 s at maximum speed and centrifuged for 30 min at 10,000 *g*. An equal volume of isopropanol was added to each supernatant, and the sample mixed, incubated at −20 °C for 1 h, and centrifuged for 20 min at 4 °C and 10,000 *g*. The resulting pellets were washed with 70% ethanol, dried, and, finally, resuspended in 300–500 μL sterile distilled H_2_O.

### 4.2. Strategy for PCR Amplification of Overlapping Genomic DNA Fragments

For sequencing *E. ocellatus* genes encoding PII-SVMP EOC00006 [Q14FJ4] and PI-SVMP EOC00028 [Q2UXQ3] we employed a similar iterative process as described in [47]. Full-length cDNA-deduced amino acid sequences of disintegrin domains [103] and of the genomic organization of dimeric disintegrin domains [AM286800] [87] and PIII-SVMP EOC00089 [47] from the same species were used as templates to design primers for the PCR-amplification of protein-specific genomic sequences (Table 2).

PI-SVMP stretch ^72^AREILNS.....QRWNDLQ^263^ was amplified on an Eppendorf Mastercycle^®^ epgradient S instrument in a 50 μL reaction mixture containing 17.5 μL of H_2_O, 25 μL Master-Mix (Thermo Scientific, Waltham, MA USA) including buffer, dNTPs, and Phusion High-Fidelity DNA polymerase, 2.5 μL of each primer (10 μM) Met1PIRv and Met5PIFw, 1.5 μL of DMSO (100%), and 1 μL of genomic DNA (50 ng/μL). PCR conditions included an initial denaturation step at 98 °C for 30 s followed by 35 cycles of denaturation (20 s at 98 °C), annealing (15 s at 63 °C), extension (300 s at 72 °C), and a final extension for 5 min at 72 °C. All other PCR amplifications were carried out in the same thermocycler using iProof High Fidelity polymerase (BioRad, Hercules, CA, USA). The 50 μL reaction mixture contained 10 μL of 5×buffer, 1 μL of 10 mM (each) dNTPs, 2 μL of MgCl_2_ 50 mM, 1.5 μL of DMSO (100%), 1 μL of each Fw and Rv primer (10 μM), 1 μL of genomic DNA (50 ng/μL), and 32.5 μL of water. PCR conditions included an initial denaturation step at 98 °C for 120 s followed by 35 cycles of denaturation (10 s at 98 °C), annealing (15 s at the lower melting temperature of the primers), extension (60 s per Kb at 72 °C), and a final extension for 5 min at 72 °C.

### 4.3. Purification and Cloning of PCR Products

PCR-amplified DNA fragments were purified from agarose electrophoretic bands using the GENECLEAN Turbo kit (MP Biomedicals). The purified fragments were inserted into pJET_1.2 (Thermo Scientific, Waltham, MA USA) using phage T4 ligase and cloned into *E. coli* DH5α by electroporation at 1700 V. Transformed cells, resuspended in 200 μL LB medium, were incubated at 37 °C for 1 h, and were subsequently plated on LB agar/ampicilline to select positive clones. The presence of the inserted DNA fragments was verified by PCR amplification or digestion of the expression vector with the restriction enzyme Bgl II. The inserted DNA fragments were sequenced in-house on an Applied Biosystems model 377 DNA sequencing system (Foster City, CA, USA) using pJETFw and pJETRv primers.

### 4.4. Sequence Analysis

Exon-intron boundaries were localized by visual inspection and corroborated using Wise2 [104]. Amino acid and nucleotide sequence similarity searches were done using BLAST [105]. Multiple sequence alignments were performed using ClustalW2 [106]. The occurrence of retrotransposable elements and simple nucleotide sequence repeats (SSRs) were assessed using RepeatMasker (version rm-20110920) [107], a program that screens DNA sequences for interspersed repeats and low complexity DNA sequences included in the Repbase database [108].

### 4.5. Sequence Availability

Pre-pro EOC00006-like PII-SVMP and EOC00028-like PI-SVMP gene sequences have been deposited with the NCBI GeneBank [109] and are accessible under accession codes KX219964 and KX219965, respectively.

## Figures and Tables

**Table 1:** Comparison of type and location of retroelements identified in introns of *E. ocellatus* PII-SVMP EOC00006-like and PI-SVMP EOC00028-like genes.

Intron	PII-SVMP	PI-SVMP
	Inserted Retroelement	
1	SINE/Sauria	2 SINE/Sauria, LTR/ERV1, DNA/hAT-Ac
3	LINE/L2/CR1	LINE/L2/CR1
5	LINE/L2/CR1	LINE/L2/CR1
6	SINE/Sauria	-
8	LINE/L2/CR1	-
9	-	SINE/Sauria
10	DNA transposon	DNA transposon

**Table 2:** Forward (Fw) and reverse (Rv) primers used to PCR-amplify genomic DNA stretches from *E. ocellatus* PII-SVMP EOC00006-like (**right**) and PI-SVMP EOC00028-like (**left**) genes.

Primer	DNA sequence	Primer	DNA sequence
Sp35_Eo Fw	ATGATCCAAGTTCTCTTGGTAACTATATGCTTAGC	5’ PS-Disi Fw	ATGATCCAAGTTCTCTTGG
Met14PI Fw	CTATATGCTTAGCAGTTTTTCCATATC	Intr4 Fw	ATGACACTGACCTCTAGAGTTGG
Intr1F1PI Fw	CTAGTCATTCCGGCCATATGAC	IntrB9_4-2 Fw	AAGCTTGCTTGCTAGTAGGTGG
Intr2F1PI Fw	ATCAGTCTGAGAGGATGCATTTCC	Intr4 Rv	TGGACATTGTATGGTCACCTG
Intr3F1PI Fw	GTGACCATGCAATGTCCATATG	Prodom 3 Fw	GGAGCTTTTAAGCAGCCAGAG
Met15PI Fw	GTTGCCTGTAGGAGCTGTTAAG	Prodom 3 Rv	CTCTGGCTGCTTAAAAGCTCC
Prodom 2 Fw	GACGCTGTGCAATATGAATTTG	Prodom 2 Fw	GACGCTGTGCAATATGAATTTG
Prodom 2 Rv	CAAATTCATATTGCACAGCGTC	Prodom 2 Rv	CAAATTCATATTGCACAGCGTC
Intr3 Rv	GCACCAACTCTGTATCTCAGTC	Intr3 Fw	CACAGGTAAATAAGCCACAAACACC
Pro2 Fw	CAGTGAGACTCATTATTCCCCTGATGGCAG	Intr3 Rv	GCACCAACTCTGTATCTCAGTC
Pro3 Rv	CTGCCATCAGGGGAATAATGAGTCTCACTG	Pro2-SVMP_Fw	CAGAAGATTACAGTGAGACTCATTATTCCCWGATGG
IntrB13-1 Fw	CTTGCCTCCCTATAGGATCACTGC	Pro3-SVMP_Rv	CTGCCATCAGGGGAATAATGAGTCTCACT
Met16PI Rv	GATGCGTCCATAATAATAGCAGTG	IntrB13-1 Fw	CTTGCCTCCCTATAGGATCACTGC
Prodom 1 Fw	GATGCCAAAAAAAAGGATGAGG	Prodom 1 Fw	GATGCCAAAAAAAAGGATGAGG
Prodom 1 Rv	CCTCATCCTTTTTTTTGGCATC	Prodom 1 Rv	CCTCATCCTTTTTTTTGGCATC
IntronB7PI Fw	TGGAACAACAGCTGTTGTTATGACG	Intr2 Fw	ACAATGGGAAACTGAGGAACAG
IntronB7PI Rv	TGAGAGACATGCTGATGTGGTC	Intr2 Rv	GGGAACTCTGACTTAGAGAAAGTC
Met4 PI Fw	GACCCAAGATACATTCAGCTTGTC	Met1PII Fw	CAACAGCATTTTCACCCAAGATAC
Met4 PI Rv	GACAAGCTGAATGTATCTTGGGTC	Met1PII Rv	GTATCTTGGGTGAAAATGCTGTTG
Met8PI Rv	TATCCATGTTGTTATAGCAGTTAAATC	Met 1-2 Fw	CATGGATACATCAAATTGTCAACG
Intron B16 Fw	TGTGCTTACCCAACACTGAGCC	Met 1-3 Rv	TGTACATCTGTCAGGTGGACATG
Met5 PI Fw	GCACGTGAAATTTTGAACTCA	Met2PII Fw	GCCGTTCACCTTGATAACCTTATAGG
Met5PI Rv	GAGTTCAAAATTTCACGTGCTG	Met2PII Rv	CCTATAAGGTTATcAAGGTGAACGGC
Met9PI Rv	AGCATTATCATGCGTTATGCG	Met 6 PII Fw	CCACAATCGTCTGTAGCAATTACTGA
Met3 PI Fw	GGAAGAGCTTACATGGAGAG	Met 6 PII Rv	TCAGTAATTGCTACAGACGATTGTGG
Met3PI Rv	CTCTCCATGTAAGCTCTTCC	Met3 PII Fw	GATCATAGCACAGATCATCTTTGG
Met2PI Rv	GCTCCCCAGACATAACGCATC	Met3PII Rv	CCAAAGATGATCTGTGCTATGATCc
IntrB23PI Fw	CTGACTATGACTCACTTAACAACTGG	Met 4 Fw	ATGATCCAGGTTCTCTTGGTAACTATATG
IntrF2PI Fw	GGCCGCGTGAATGCATCTGCTTC	Met 4 Rv	TGAACTGATAGGAACGGTATTGTG
Intr2F2PI Fw	GCATCAGTTTGTTCGCACTCAATAAAG	Fw_Ocella NcoI	ATCCATGGTAGACTGTGAATCTGGACC
Intr3F2PI Fw	GAGCATAATCTGGAACTAAGATCAAG	IntrDis1 Rv	ATACGGCTAGTATGGAGCAGG
Met7PI Fw	GCACAAGATTCCTATCACTTCAG	Dis PII Rv	TCACATCAACACACTGCCTTTTGC
Met13PI Rv	TCCTACCTGCAAAAGTTCATTTTC	-	-
Intron B10PI Rv	CTGACTCAGGGCACCAATCTC	-	-
Met1PI Rv	CTACTGCAGATCGTTCCATCTCTG	-	-

## Supplementary Materials

The following are available online at www.mdpi.com/2072-6651/8/7/216/s1, Figure S1: Pairwise nucleotide sequence alignments of topologically equivalent paralog introns 1–12 from Pre-pro EOC00006-like PII-SVMP and 1–13 from Pre-pro EOC00028-like PI-SVMP gene sequences.

## Appendix A

Genomic sequence of *E. ocellatus* EOC00006-like PII-SVMP gene. The locations and identities of the primers used to PCR-amplify genomic sequences (listed in Table 2) are indicated. Protein-coding DNA regions are in upper letters and boldface, and the encoded amino acid sequence is displayed below the DNA sequence. Start of introns are labelled EoPII-X, where “X” corresponds to intron number. The beginning and the signal peptide, propeptide, metalloproteinase and the short-disintegrin domains are specified. Numbers at the right correspond to amino acid numbering of the DNA-deduced pre-pro-PII-SVMP relative to the mature SVMP. The *N*-terminal glutamine of the metalloproteinase domain has been assigned residue 1. The extended Zn^2+^-binding environment (HEXXHXXGXXH) and the RGD integrin inhibitory motif stand on yellow background. The only two amino acids (−70I/V, and −111H/R) that distinguish this sequence from that of PII-SVMP EOC00006 (Q14FJ4) are shown in bold and red. The remains of a disintegrin-like domain transformed into intron EoPII-12 are underscored in italics and on cyan background. SINE/Sauria, LINE/L2/CR1 and DNA transposon retroelements are highlighted on a gray background. Simple sequence repeats (SSR, microsatellites) are shown in light green background.

Genomic sequence of *E. ocellatus* EOC00028-like PI-SVMP gene. The locations and identities of the primers used to PCR-amplify genomic sequences (listed in Table 2) are indicated. Protein-coding DNA regions are in upper letters and boldface, and the encoded amino acid sequence is displayed below the DNA sequence. Start of introns are labelled EoPI-X, where “X” corresponds to intron number. The beginning and the signal peptide, propeptide, metalloproteinase domains and the *C*-terminal extension are specified. Numbers at the right correspond to amino acid numbering of the DNA-deduced pre-pro-PII-SVMP relative to the mature SVMP. The extended Zn^2+^-binding environment (HEXXHXXGXXH) stands on yellow background. The only two amino acids (-124T/A and 15T/A) that distinguish this sequence from that of PI-SVMP EOC00028 (Q2UXQ3) are shown in bold and red. The remains of a disintegrin-like domain and a dimeric disintegrin domain transformed into intron EoPI-12 are underscored in italics and on cyan background. The *N*-terminal glutamine of the metalloproteinase domain has been assigned residue 1. SINE/Sauria, LINE/L2/CR1, LTR/ERV1, DNA/hAT-Ac and DNA transposon retroelements are highlighted on a gray background. Inserted nucleotide sequences in introns 1 (582 nucleotides between positions 1534–1582, including a SINE/Sauria element); 9 (between nucleotides 194–195 of the topologically equivalent intron of PII); 11 (replacing nucleotides 1-66 of PII intron 11 for a stretch of 3281 nucleotides); and 12 (after nucleotide 999 of the homologous PII intron) are underlined. Simple sequence repeats (SSR, microsatellites) are shown in light green background.
