# Supplementary material for: Insights into the Evolution of a Snake Venom Multi-Gene Family from the Genomic Organization of Echis ocellatus SVMP Genes
Source: Toxins (Basel). 2016 Jul 12;8(7):216. doi: 10.3390/toxins8070216 (PMC4963849; doi:10.3390/toxins8070216)
Supplement: Supplementary file 1 [file toxins-08-00216-s001.pdf]

Supplementary Materials: Insights into the Evolution of a Snake Venom Multi-Gene Family from the Genomic Organization of *Echis ocellatus* SVMP Genes

Libia Sanz and Juan J. Calvete

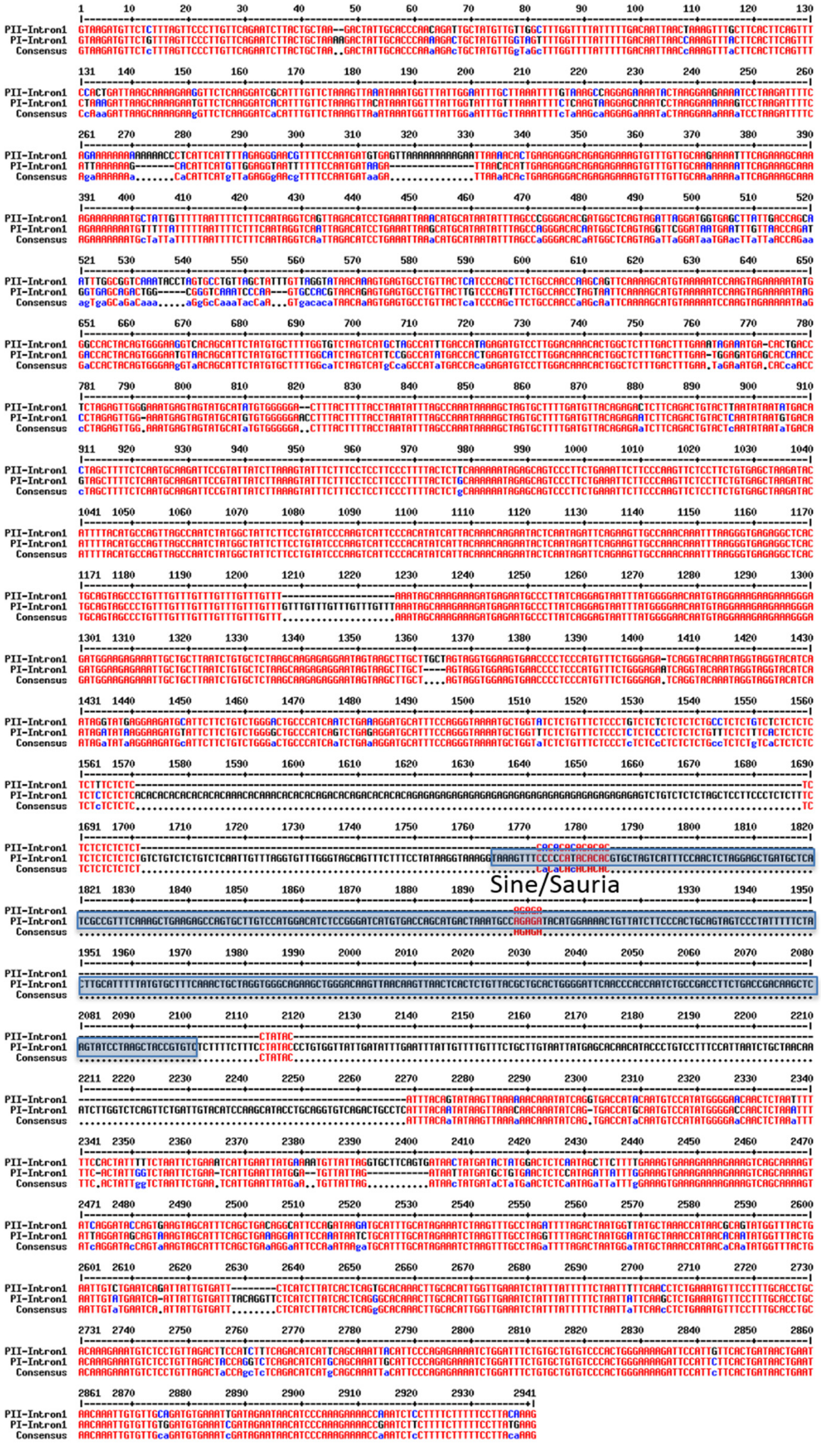

(A)

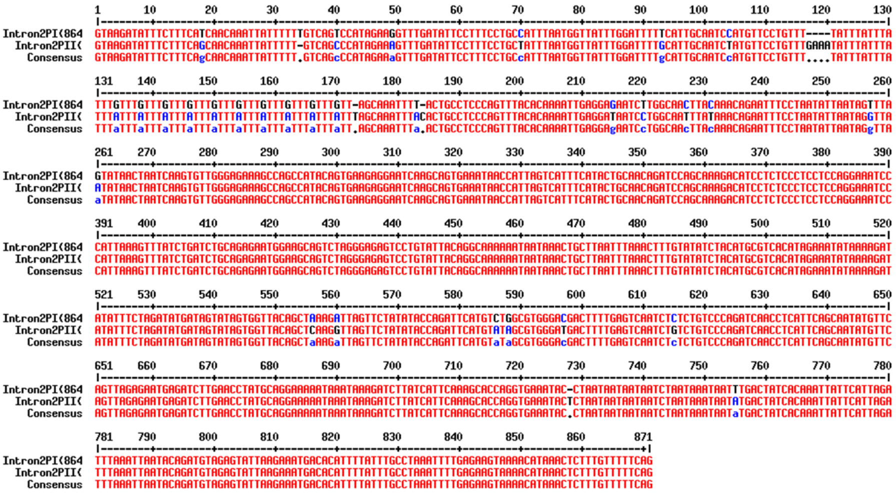

(**B**)

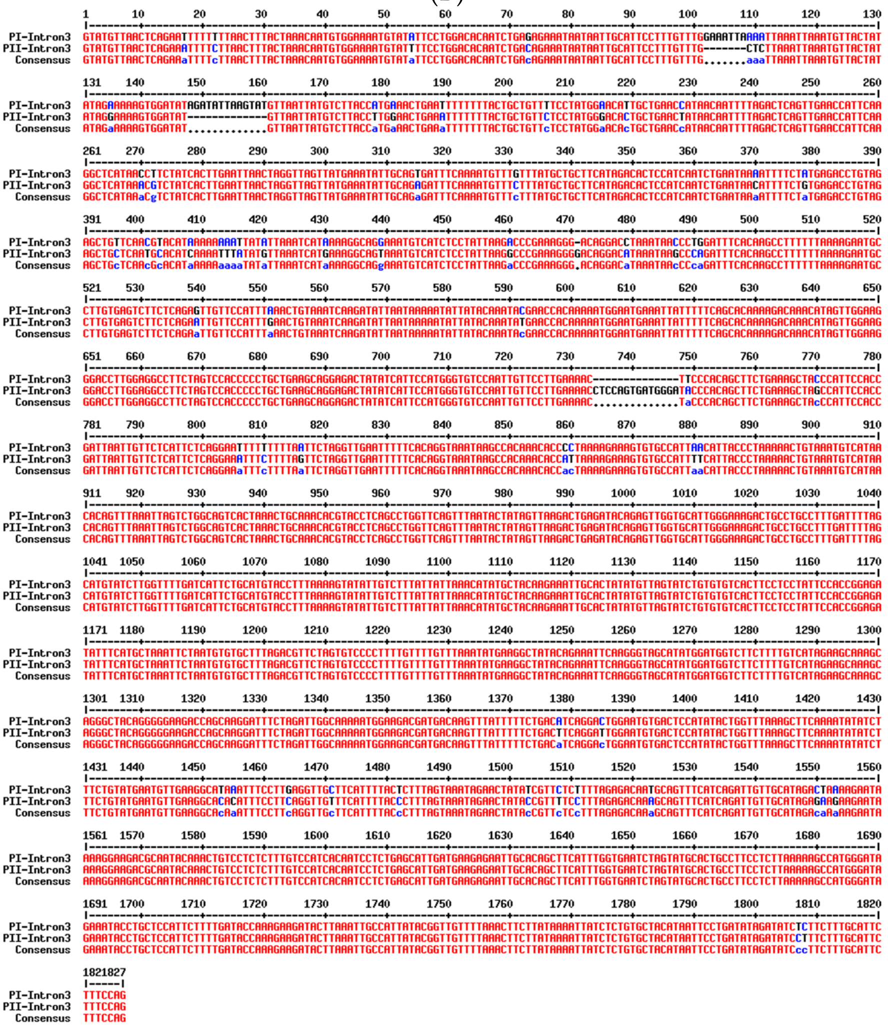

(**C**)

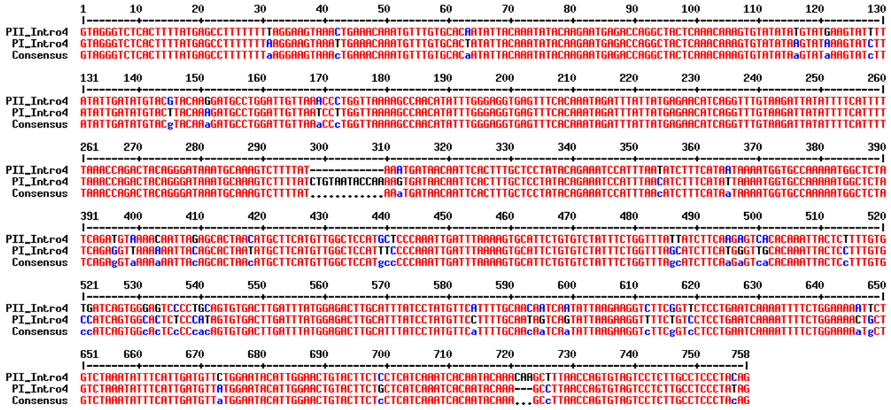

(**D**)

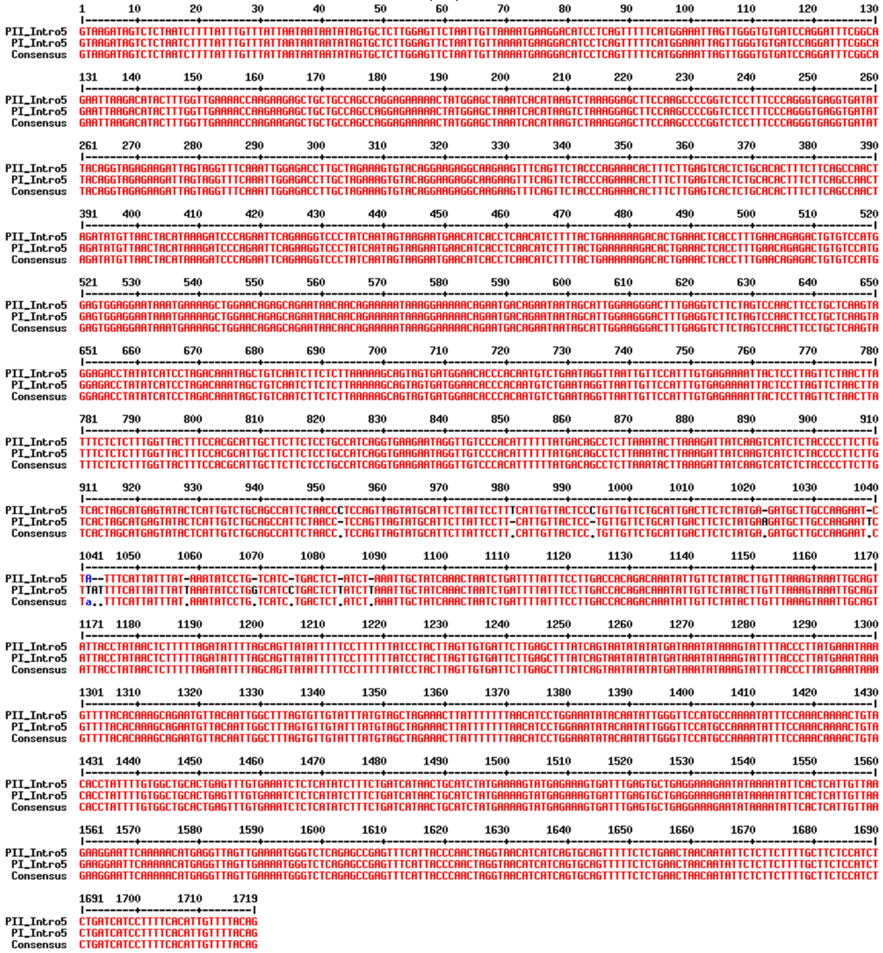

(**E**)

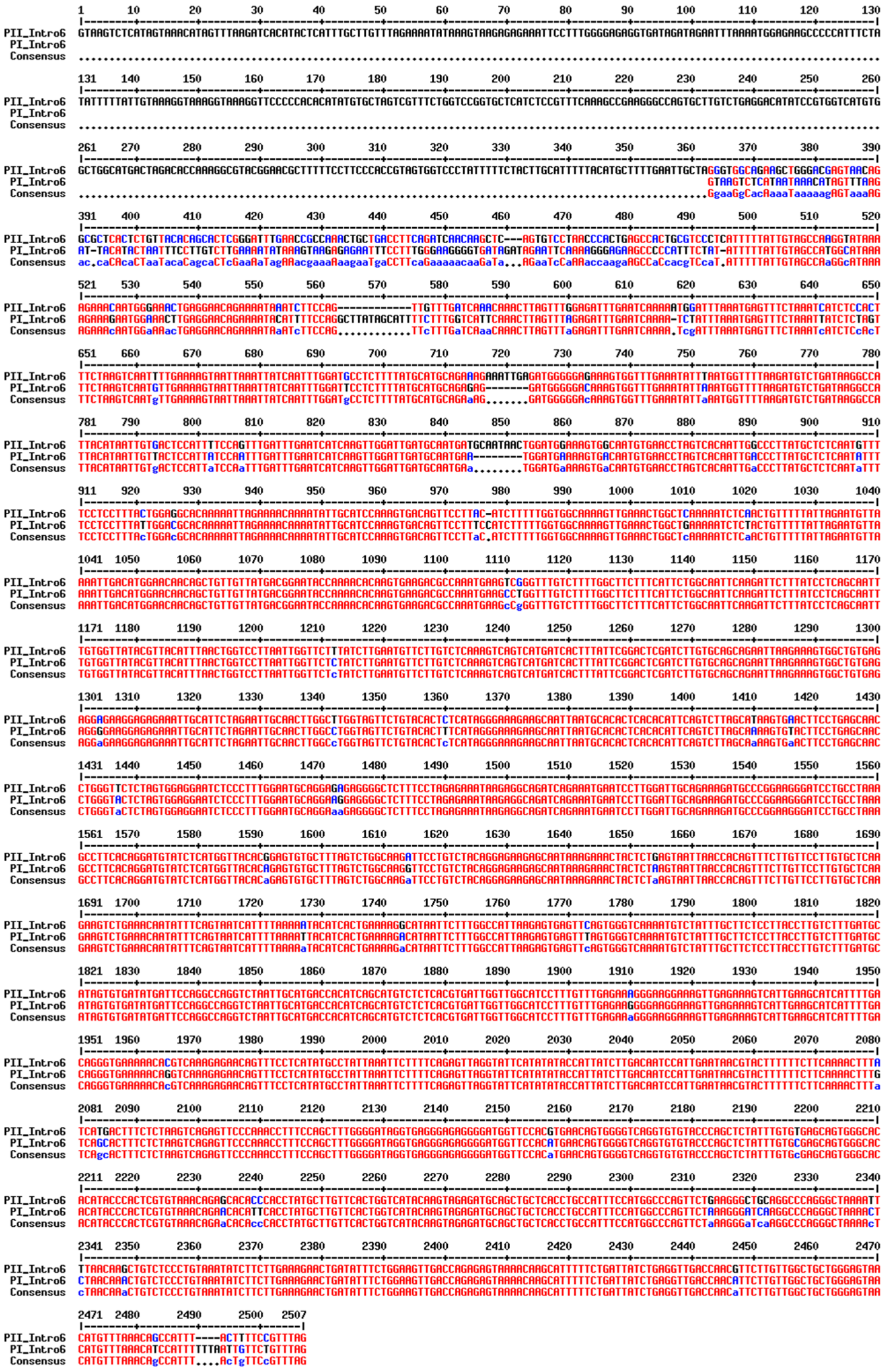

(**F**)

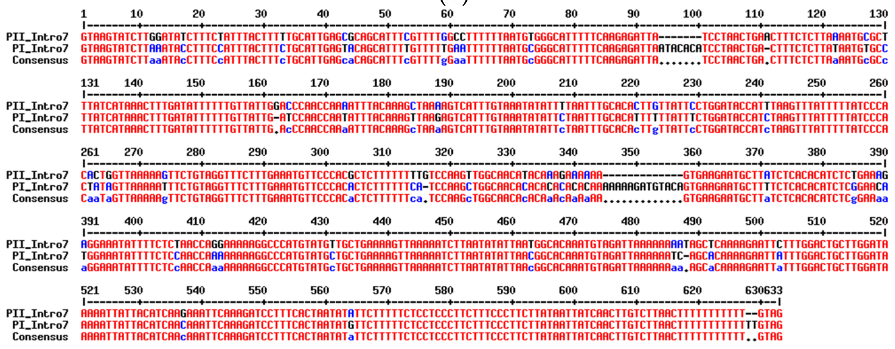

(**G**)

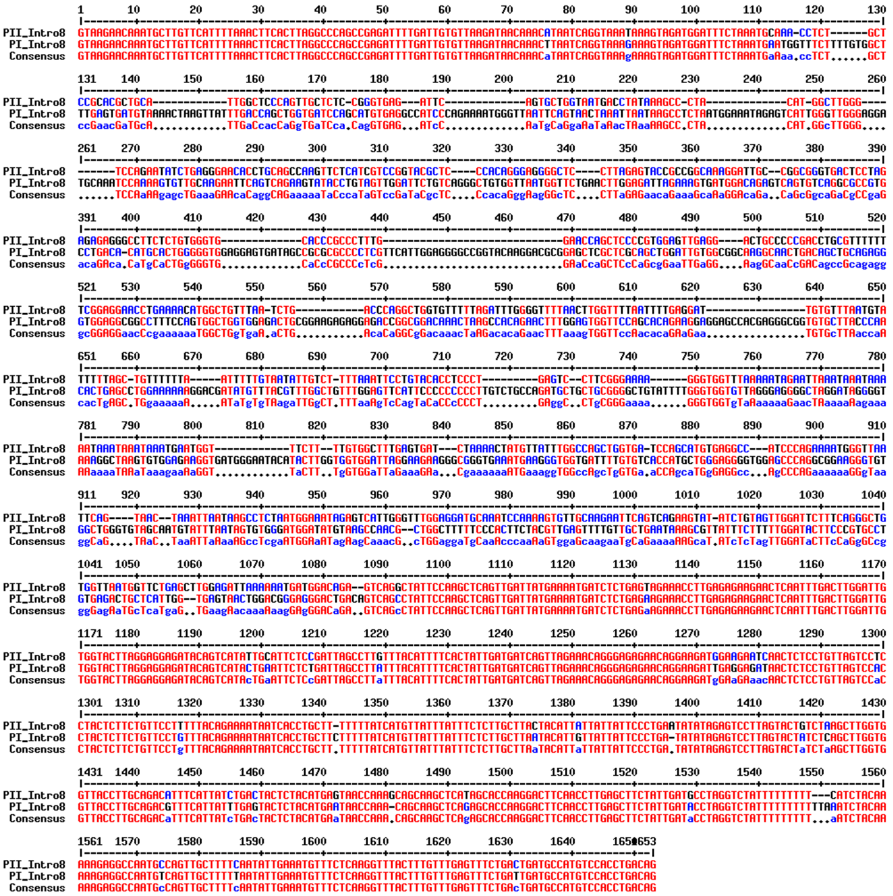

(H)

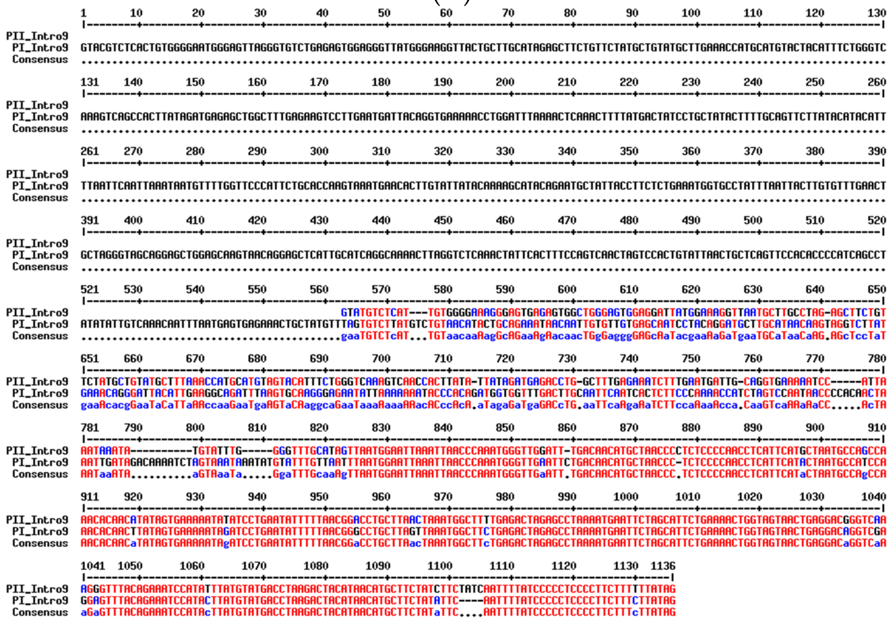

(I)

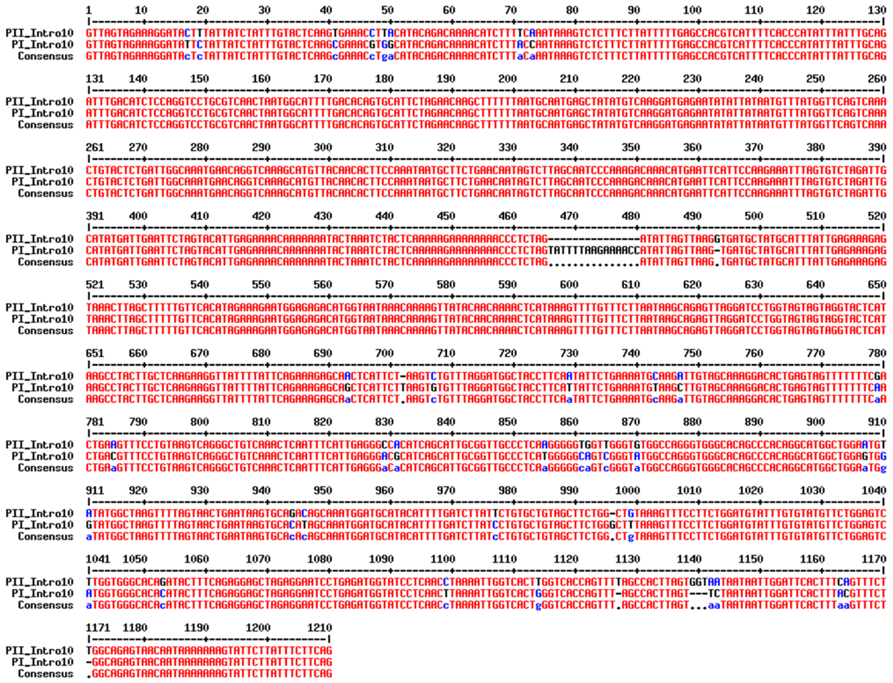

(J)

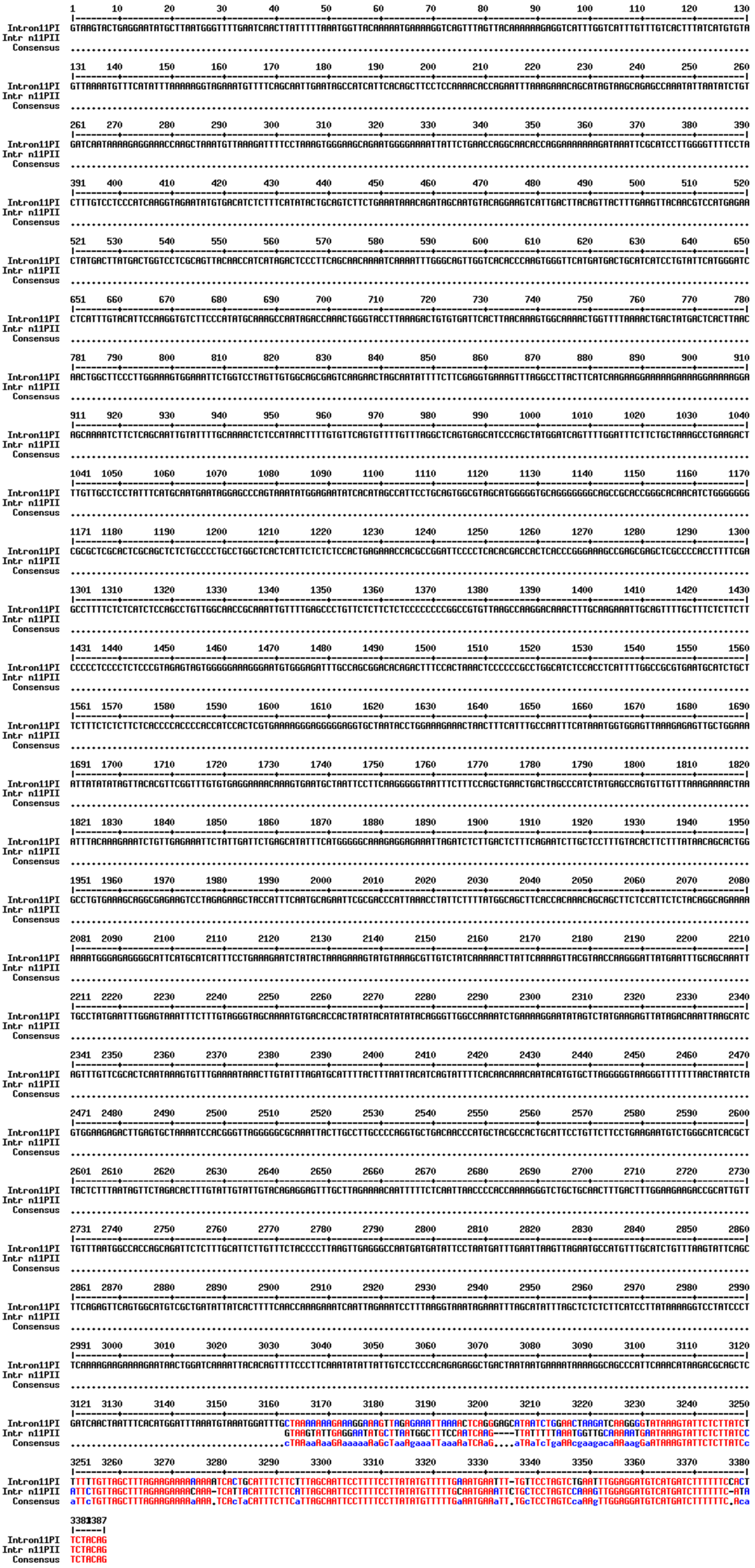

(**K**)

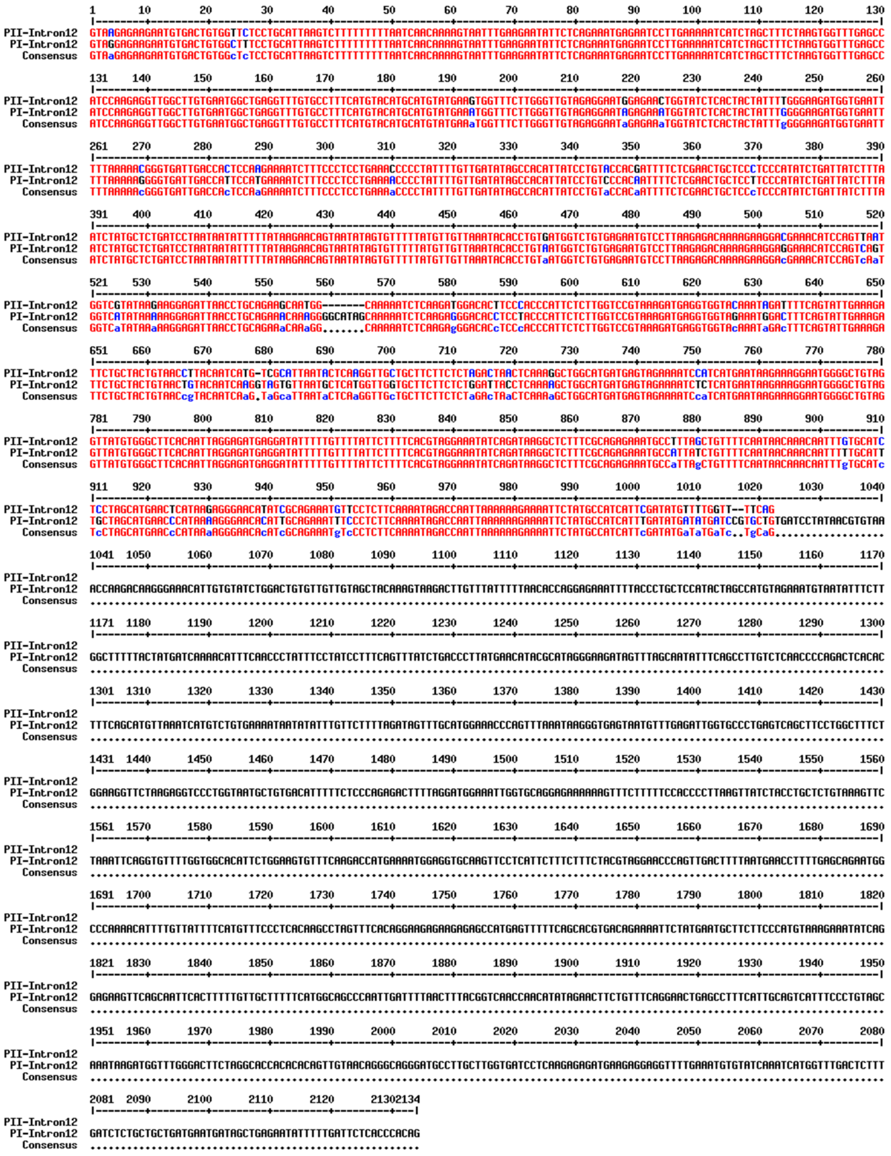

(**L**)

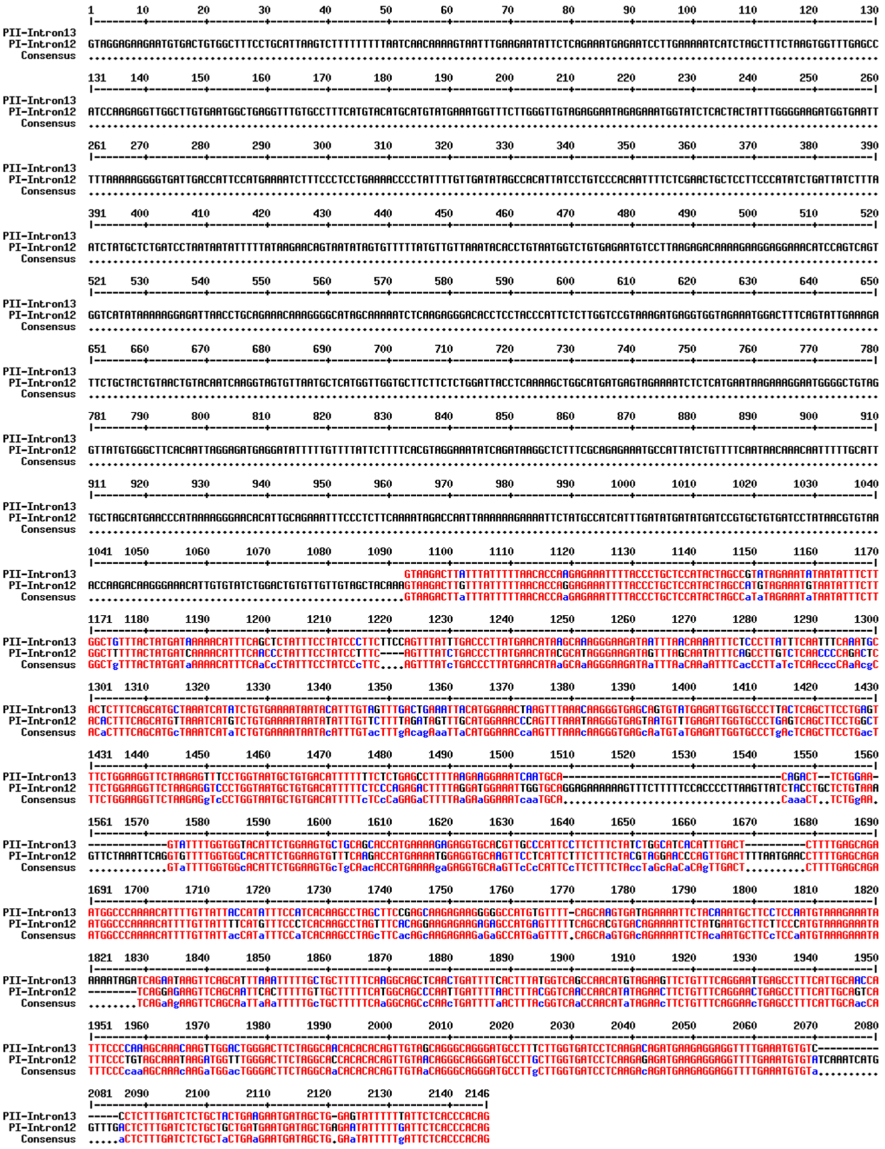

(**M**)

Figure S1: Pairwise nucleotide sequence alignments of topologically equivalent paralog introns 1–12 from Pre-pro EOC00006-like PII-SVMP and 1–13 from Pre-pro EOC00028-like PI-SVMP gene sequences.
